# Hydrothermal synthesis of novel 1-aminoperylene diimide/TiO_2_/MoS_2_ composite with enhanced photocatalytic activity

**DOI:** 10.1038/s41598-020-78894-y

**Published:** 2020-12-15

**Authors:** Yongshan Ma, Yue Wang, Tianyi Jiang, Fengxia Zhang, Xuemei Li, Yanyan Zhu

**Affiliations:** 1 School of Municipal and Environmental Engineering, Shandong Jianzhu University, Shandong, 250101 People’s Republic of China; 2 Shandong Provincial Key Laboratory of Metrology and Measurement, Shandong Social Justice Institute of Metrology, Shandong Institute of Metrology, Jinan, 250014 People’s Republic of China

**Keywords:** Environmental sciences, Materials science

## Abstract

1-aminoperylene diimide/TiO_2_/MoS_2_ composite (NH_2_-PDI/TiO_2_/MoS_2_) with ordered structure was prepared by hydrothermal synthesis method. The composite was characterized by XRD, SEM, FTIR, XPS, BET, DRS, PL, EIS, Raman, photocurrent, and Mott-Schottky plots spectroscopy. The potential positions of the conduction and valence bands, and the band gap energy of the semiconductors were estimated. The composite exhibited higher photocatalytic activity compared with the mono-component systems. The apparent rate constants (k) were determined as 0.00616, 0.00352, 0.00738, 0.00517, 0.00752, and 0.00806 min^−1^ for TiO_2_, NH_2_-PDI, NH_2_-PDI/TiO_2_, MoS_2_, MoS_2_/TiO_2_, and NH_2_-PDI/TiO_2_/MoS_2_, respectively. The detection of radical scavengers confirmed that superoxide radicals, photogenerated holes, and photogenerated electrons were the main active substances for MB degradation. Between type II- heterojunction mechanism and Z-scheme mechanism, the latter could explain the enhanced photocatalytic activity of the composite better. The Z-scheme mechanism accumulates more electrons at CB level of NH_2_-PDI and hence generates more super oxide radicals.

## Introduction

Water pollution is an important issue of concern in many countries. Many dyes in textile printing or dyeing wastewater contain aromatic compounds, which are chemically stable and harmful to human health^1^. Because of the adverse environmental impacts of these refractory organic compounds, it is necessary to develop new methods to degrade them. Photocatalysis is a promising technique for photodegradation of hazardous chemicals in wastewater^2^. Titanium dioxide (TiO_2_) and TiO_2_ based nanostructures are being used for photocatalytic degradation of organic pollutants to protect environment^3^. With the advantages of low cost, large specific surface area, high oxidizing power, and good chemical stability, TiO_2_ has become one of the most promising candidates for photocatalysis^4^. However, the catalytic performance of TiO_2_ is severely limited by the large band gap (3.2 eV), low photon utilization rate of solar energy (about 5%), and high recombination rate for photogenerated electron–hole pairs^5,6^. On the other hand, the industrial treatment of wastewater containing various organic pollutants using TiO_2_-photocatalyst is not common due to low efficiency of photodegradation^7^. To solve this problem, several methods have been used to improve the photocatalytic efficiency of TiO_2_. It has been reported that in a suitable heterostructured system, the presence of heterojunction changes the energy band positions and their inclination on the interface to accelerate the migration of photogenerated charge carriers, and eventually enhance the efficiency of photocatalyst^8^.

Perylenetetracarboxylic acid diimides (PDIs) are cheap organic dyes with high photothermal stability and strong absorption in the visible region^9,10^. As a typical n-type semiconductor, PDIs have high electron mobility and electron affinity [LUMO] due to its strong π–π stacking between the conjugated π bonds^11,12^. PDIs materials have been used in photocatalysis because it can improve the light absorption and decrease the photo-generated electron–hole recombination^13^. PDIs have been introduced into TiO_2_, ZnO, and other photocatalysts to improve the photocatalytic performance^14,15^. However, such composite materials still have some defects such as quick recombination of photoexcited electron–hole pairs and photoetching, which lead to low efficiency of visible light driven photodegradation^16^. Therefore, it is necessary to further improve their photocatalytic activities and stabilities.

Molybdenum disulfide (MoS_2_) is a rapidly rising two-dimensional layered material, which has aroused enormous scientific interest in developing new MoS_2_-based materials for rich potential applications^17^. MoS_2_ with different structures such as nanoribbon and nanosheet has been successfully synthesized^18,19^. It has unique physical properties that distinguish it from other materials and has been used in many applications such as lubricant, photovoltaic, and photocatalysis^20,21^. Hu et al. synthesized bulk, nano-slice, and nano-ball MoS_2_ by precipitation method, and found that the photocatalytic efficiency of nano-slice MoS_2_ towards methyl orange (90%) was higher than others^22^. James et al. synthesized MoS_2_ nanoparticles through thermally decomposing method, and the material was used for photocatalytic degradation of methylene blue (MB) with an efficiency of 30%^23^. Xu et al. prepared flower-like MoS_2_ nanopowders through hydrothermal method for photocatalytic degradation of Rhodamine B (RhB) and an efficiency of 15% was achieved^24^. In addition, the good conductivity of monolayer MoS_2_ can efficiently separate the electron–hole pairs and enhance the photocatalytic activity. Hence, MoS_2_ can be used in TiO_2_ photocatalyst to improve its photocatalytic performance. For instance, Behzad Pourabbas et al. reported the photo-oxidation of phenol by MoS_2_/TiO_2_ hybrids. Suddhasatwa Basu et al. demonstrated the ability to degrade RhB by MoS_2_/TiO_2_ nanocomposite^25^. Giri et al. reported the photo-oxidation of RhB by MoS_2_/TiO_2_ hybrids^26^. Du et al. reported a MoS_2_/CdS/TiO_2_ photocatalyst which has high photocatalytic activity and stability^27^. This technique produces powerful non-selective oxidants, such as superoxide radicals and hydroxyl radicals, which can degrade and mineralize a wide variety of pollutants^28^. The produced electrons-holes would recombine in pure TiO_2_, which limited its application in the photodegradation. By preventing electron/hole recombination and semiconductors aggregation, multi-component support can improve the photoreactivity of the obtained composite^29^.

**Scheme 1:**
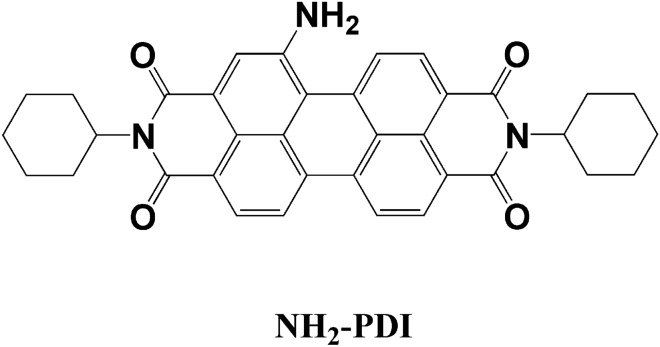
Molecular structure of 1-aminoperylene diimide (NH_2_-PDI).

In this work, a novel ternary photocatalyst: NH_2_-PDI/TiO_2_/MoS_2_ was synthesized by hydrothermal method (Scheme 1). The CB/VB (conduction band/valence band) of TiO_2_, NH_2_-PDI, and MoS_2_ are evaluated to be − 1.26/1.72 eV, − 1.56/0.8 eV, and -0.75/1.00 eV, respectively. These matched potentials are suitable for rapid charge separation between these semiconductors. The composite showed enhanced activity in the photodegradation of Methylene Blue (MB) and amoxicillin (AMX).

## Experimental

### Materials and methods

All other chemicals were analytically pure, purchased from commercial sources and used without further purification. Distilled water was used throughout the experiment. FT-IR spectrum has been studied by a Bruker Tensor-27 spectrophotometer. The ^1^H NMR and mass spectrum of NH_2_-PDI were measured on a Bruker Advance 400 spectrometer and a Bruker Maxis UHR-TOF mass spectrometer, respectively. Scanning electron microscopy (SEM) images were obtained on a Sigma, Zeiss microscope equipped with an energy dispersive X-ray (EDX) spectrometer. The specific surface area of the catalyst has been analyzed using nitrogen adsorption at 77 K applying the Brunauer–Emmett–Teller (BET) method using a micrometrics ASAP 2020 V3.00 H. X-ray diffraction (XRD) measurements have been performed using a Rigaku R-AXIS RAPID X-ray diffractometer. Crystallinity and phase composition of the as-synthesized composite have been confirmed from the Lab Ram HR800, Jobin Yvon micro-Raman measurement. The ultraviolet–visible diffuse reflectance absorption spectra (DRS) have been measured on a Shimadzu model UV–Vis diffuse reflectance spectrometer. X-ray photoelectron spectroscopy (XPS) has been performed on a KRATOS model XSAM800 instrument. Fluorescence measurements were performed at a Hitachi FL-4500 spectrometer with 290 nm excitation wavelength at room temperature. The Mott-Schottky plots, photocurrent, and electrochemical impedance spectroscopy (EIS) were measured on a CHI760E electrochemical workstation using a three-electrode system. Platinum wire as counter electrode, ITO deposited by photocatalyst was used as working electrodes (50 mg of photocatalyst was dispersed in 2 mL of ethanol and grounded for 20 min, then it was uniformly dropped onto the FTO glass and stay still overnight to dry off the ethanol.), saturated calomel as reference electrode, and 0.1 M Na_2_SO_4_ aqueous solution was used as electrolyte. The EIS was performed at an open circuit potential at a frequency of 0–10,000 Hz, and the photoelectric response of the sample was measured at 0.0 V. Photoelectrochemical properties were measured with a 300 W xenon lamp as the light source.

### Synthesis and characterization of NH_2_-PDI

The compound NH_2_-PDI was synthesized according to literature method^30^. The synthetic route along with the characterization data of NH_2_-PDI (Fig. S-1) are reported in the Supplementary Methods.

### Preparation of TiO_2_ and NH_2_-PDI/TiO_2_

The NH_2_-PDI/TiO_2_ composite was prepared by the hydrothermal synthesis method. First, tetrabutyl titanate (10.0 mL) was dissolved in anhydrous ethanol (20.0 mL). Then, 5.0 mL of distilled water was slowly added under vigorous stirring. Later, NH_2_-PDI (0.01 g) was dissolved in dichloromethane (5.0 mL) and added to the solution under sonication. The resulting mixture was stirred for 12 h and then transfer to the hydrothermal kettle for 3 h at 200 °C. The precipitate was filtered, washed thoroughly with distilled water, and dried in an air oven at 100 °C for 4 h. This catalyst contained 0.1 wt% of NH_2_-PDI. Pure TiO_2_ was prepared by the same method.

### Preparation of MoS_2_

MoS_2_ was synthesized by hydrothermal route using Na_2_MoO_4_·2H_2_O and NH_2_CSNH_2_ as Mo and S sources, respectively. Briefly, Na_2_MoO_4_·2H_2_O (2.0 g) and NH_2_CSNH_2_ (8.0 g) was dissolved in 60 mL of water under vigorous stirring for 30 min. The mixture was kept in a hydrothermal kettle at 200 °C for 24 h. The resulting black precipitate was washed several times with ethanol and water to further remove impurities and contaminants followed by a centrifugation and drying process at 60 °C for 6 h to obtain MoS_2_ nanoarchitectures.

### Preparation of MoS_2_/TiO_2_ and NH_2_-PDI/TiO_2_/MoS_2_

The NH_2_-PDI/TiO_2_/MoS_2_ composite was fabricated by loading MoS_2_ onto the preformed NH_2_-PDI/TiO_2_ solid solutions. Hydrothermal synthesis of NH_2_-PDI/TiO_2_/MoS_2_ composite was prepared as follows: 3.0 g NH_2_-PDI/TiO_2_ and 0.06 g of MoS_2_ were dispersed in 5 mL water. The resulting mixture was stirred for 24 h and then transferred to a hydrothermal kettle at 200 °C for 4 h. The prepared composite material was filtered, washed thoroughly with distilled water, and dried in an air oven at 100 °C for 4 h. NH_2_-PDI/TiO_2_/MoS_2_ was formed as a gray powder. This catalyst contained 0.1 wt% NH_2_-PDI and 2.0 wt% MoS_2_. MoS_2_/TiO_2_, NH_2_-PDI/TiO_2_/1%MoS_2_, and NH_2_-PDI/TiO_2_/3%MoS_2_ were prepared using the same procedure.

### Photocatalytic activity tests

The photocatalytic experiments were carried out in a photochemical reactor (PhchemIII, Beijing China NBeT) consisting of a 500 W xenon lamp (XE-JY500). The reaction chamber is equipped with 50 mL capacity reaction glass tubes and a magnetic stirrer. The specially designed reflector was made of highly polished aluminum and a built-in cooling fan. The light exposure length is 230 mm.

Methylene Blue (MB) and amoxicillin (AMX) were selected to test the photodegradation activity of the prepared photocatalysts. First, 50 mg of synthesized sample was suspended in 50 ml of 10 mg/L MB aqueous solution. The visible light source was obtained by a 500 W xenon lamp. Before irradiation, the suspension solutions were stirred magnetically for 120 min in the dark to achieve adsorption–desorption equilibrium. The samples were withdrawn at given time intervals and the photocatalyst was removed by centrifugation. A UV–vis spectrophotometer at 664 nm and 198 nm was used to measure the absorbance of MB and AMX solutions, respectivly.

## Results and discussion

**Figure 1:**
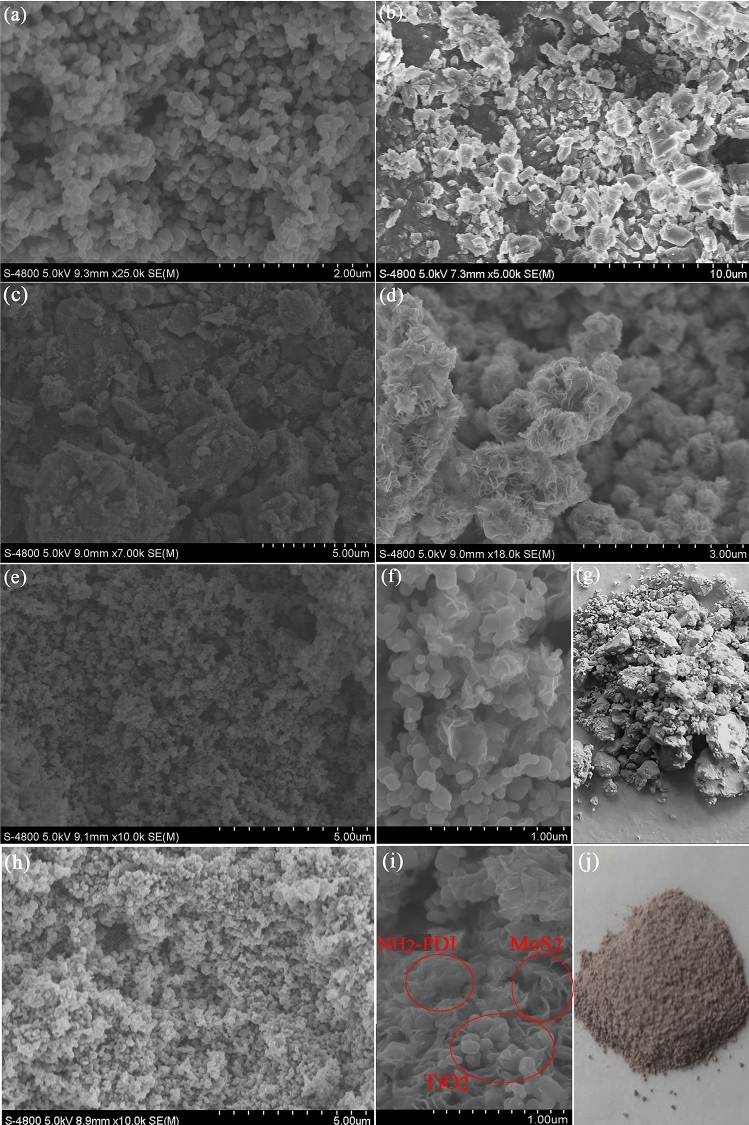
SEM images of TiO_2_ (**a**), NH_2_-PDI (**b**), NH_2_-PDI/TiO_2_ (**c**), MoS_2_ (**d**), MoS_2_/TiO_2_ (**e**,**f**), NH_2_-PDI/TiO_2_/MoS_2_ (**h**,**i**), and the appearance of MoS_2_/TiO_2_ (**g**), NH_2_-PDI/TiO_2_/MoS_2_ (**j**).

Figure 1 shows SEM images of TiO_2_, NH_2_-PDI, NH_2_-PDI/TiO_2_, MoS_2_, MoS_2_/TiO_2,_ and NH_2_-PDI/TiO_2_/MoS_2_. Figure 1(a) shows that TiO_2_ forms uniform elongated spherical particles with a length of about 300 nm and a width of about 200 nm. As can be seen from Fig. 1(b), NH_2_-PDI forms nanorods structure. The average width is 1 μm, and the length is in the range of 1–3 μm. Figure 1(c) depicts that NH_2_-PDI/TiO_2_ particles formed an agglomerated spherical structure. As shown in Fig. 1d, the average diameter of MoS_2_ nanoflowers is 0.5–1 μm. These nanoflowers have massive petals on the surfaces, which are free to gather closely. Figure 1(e) and (f) shows the morphology of the synthesized MoS_2_/TiO_2_ heterostructure characterized by SEM. It can be seen that MoS_2_ and TiO_2_ mixed together. In Fig. 1(h) and (i), a peculiar morphology of NH_2_-PDI/TiO_2_/MoS_2_ is shown. The NH_2_-PDI and MoS_2_ mixed with TiO_2_, and they can provide more active sites and mass charge transport pathways in the catalytic system. The color of MoS_2_/TiO_2_ and NH_2_-PDI/TiO_2_/MoS_2_ are shown in Fig. 1(g) and (j), respectively. MoS_2_/TiO_2_ was light grey while NH_2_-PDI/TiO_2_/MoS_2_ sample showed a red grey color.

**Figure 2:**
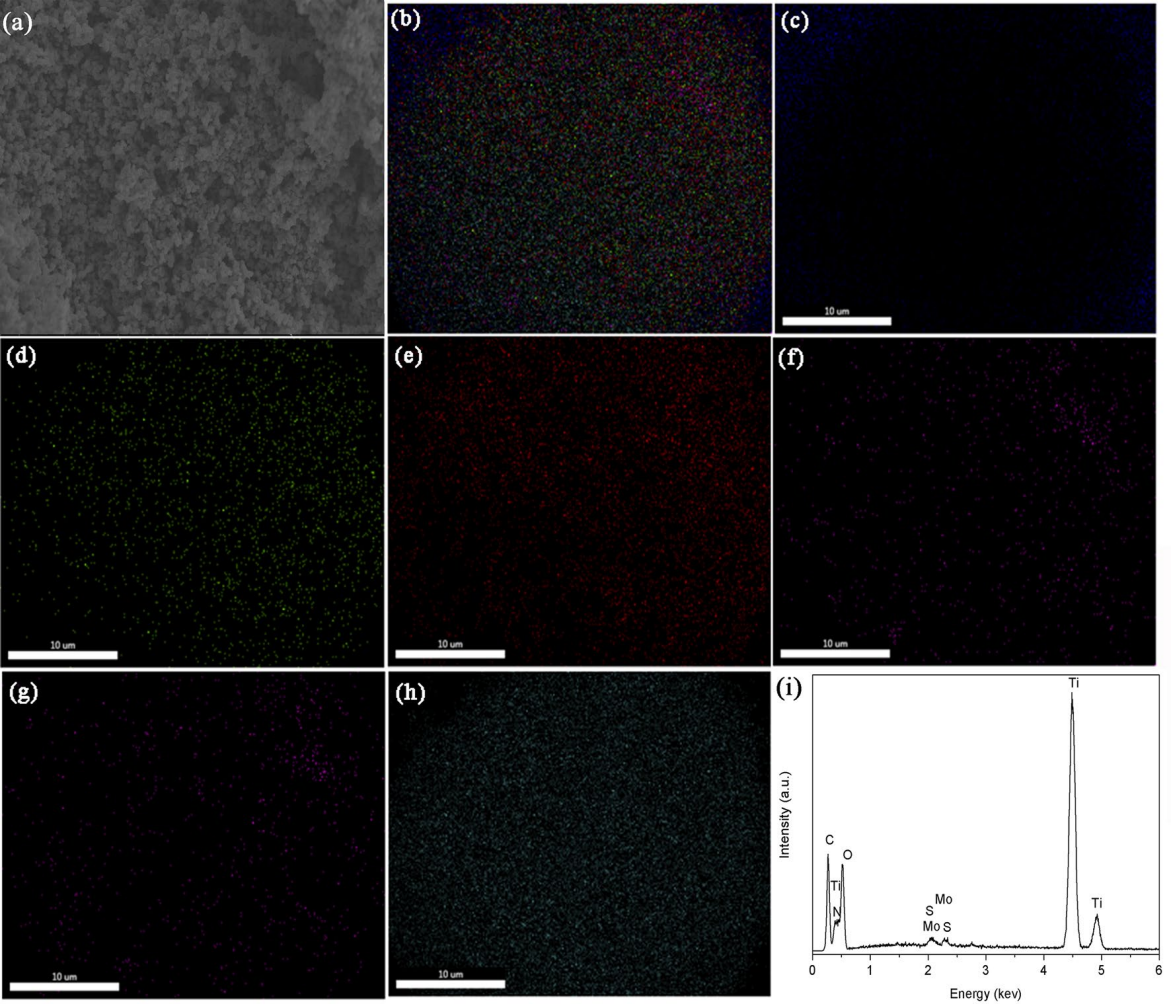
(**a**) SEM image of NH_2_-PDI/TiO_2_/MoS_2_ photocatalyst; Element mappings of Mo, S, C, N, Ti, and O elements (**b**), C (**c**), N (**d**), O (**e**), Mo (**f**), S (**g**), and Ti (**h**) in the NH_2_-PDI/TiO_2_/MoS_2_ photocatalyst; (**i**) EDX spectrum of NH_2_-PDI/TiO_2_/MoS_2_ photocatalyst.

The composition of the NH_2_-PDI/TiO_2_/MoS_2_ was further investigated by an EDX attached to SEM (Fig. 2). Figure 2(b)–(h) shows the element mappings in a selected area of the composite (Fig. 2a). The homogeneous distributions of the elements Mo, S, C, N, Ti, and O can be clearly seen from the graphs. As shown in Fig. 2(i) and Fig. S-2, EDX analysis reveals that the composite contains Ti and O (in the case of TiO_2_), Mo and S (in the case of MoS_2_), or C and N (in the case of NH_2_-PDI). The atomic ratio of Ti, Mo and C equals to 25.2: 1: 1.3, meaning that the molar ratio of TiO_2_, MoS_2_, and NH_2_-PDI is about 95.3: 1.89: 0.68. The high proportion of C is due to the elemental carbon and carbonate species adsorbed on the TiO_2_ surface.

**Figure 3:**
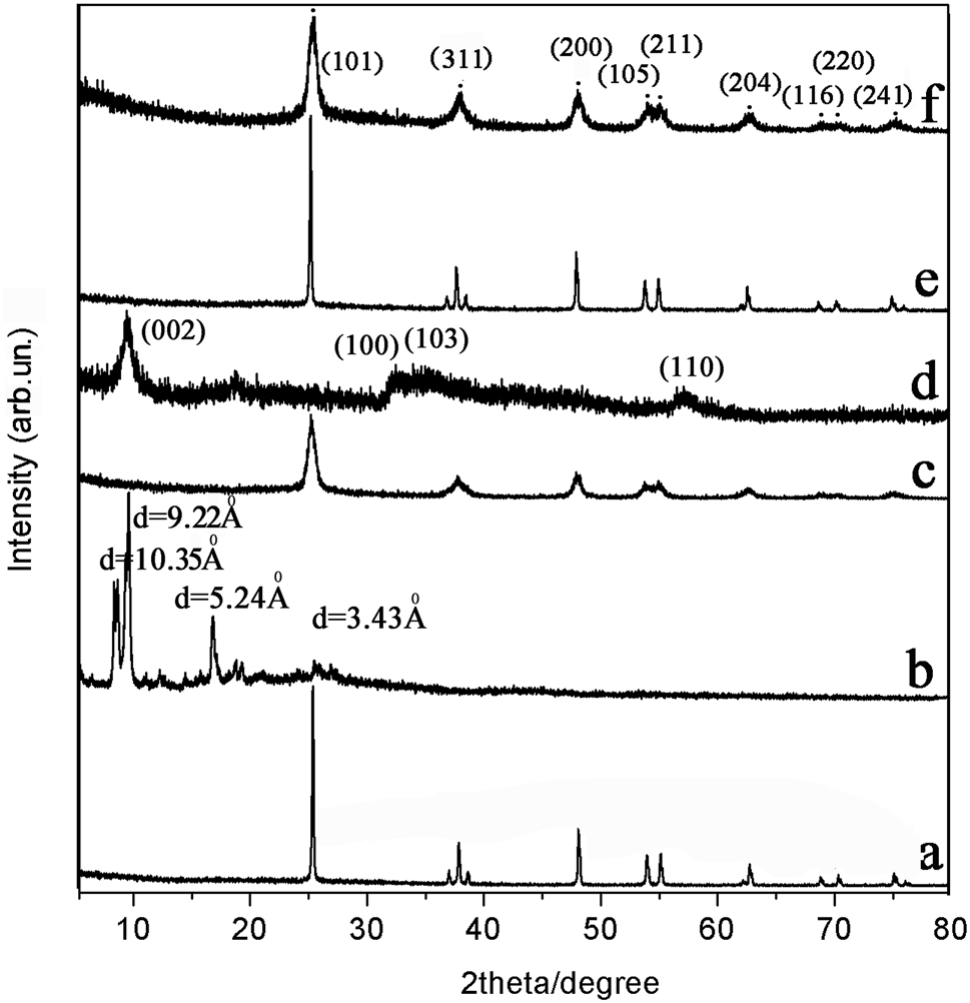
XRD patterns of TiO_2_ (**a**), NH_2_-PDI (**b**), NH_2_-PDI/TiO_2_ (**c**), MoS_2_ (**d**), MoS_2_/TiO_2_ (**e**), NH_2_-PDI/TiO_2_/MoS_2_ (**f**).

XRD measurements were performed to determine the crystalline structures of TiO_2_, NH_2_-PDI, NH_2_-PDI/TiO_2_, MoS_2_, MoS_2_/TiO_2_, and NH_2_-PDI/TiO_2_/MoS_2_ composites (Fig. 3). The crystal phase of TiO_2_ was consistent with anatase phase TiO_2_ (JCPDS NO. 71–1167) (Fig. 3a). Diffraction peaks appeared at 2θ = 25.3°, 37.7°, 48.0°, 53.8°, 55.0°, 62.6°, 58.6°, 70.6°, and 75.2°, corresponding to (101), (311), (200), (105), (211), (204), (116), (220), and (241) planes of TiO_2_, respectively^31^. The NH_2_-PDI appeared in the corresponding d-spacing as 10.35 Å, 9.22 Å, 5.24 Å, and 3.43 Å (Fig. 3b). The peak at 2*θ* = 25.74° (*d* spacing 3.43 Å) can be attributed to the π-π stacking of the adjacent NH_2_-PDI, since the distance of π-π stacking between the perylene nuclei was about 3.5 Å^32^. It was well known that PDIs exhibit a typical sharp diffraction peak at about 8–9°, which was centered at 9.57° (*d* spacing 9.22 Å)^33^. Additionally, X-ray powder diffraction measurements on NH_2_-PDI showed that the first diffraction peak appeared at 2*θ* = 8.53° (*d* spacing 10.35 Å) and the second-order diffraction peak appeared at 2*θ* = 16.85° (*d* spacing 5.24 Å). The first diffraction peak was assigned as (001) and the second peak was assigned as (002) α-form crystal diffraction, which has been reported by Miyata^34^. The multiple orders of reflection indicated that the self-assembled structures of NH_2_-PDI are well-ordered and layered microstructures. For the pure 3D flower-like spherical MoS_2_ nanostructure, the peaks correspond to (002), (100), (103), and (110) diffraction planes of 2H-MoS_2_ (JCPDS: 37-1492), which is consistent with previous studies (Fig. 3d)^35^. For MoS_2_/TiO_2_, the addition of MoS_2_ did not change the diffraction peak positions of TiO_2_ obviously, indicating that MoS_2_ was not incorporated into the TiO_2_ lattice. Obviously, both NH_2_-PDI/TiO_2_ and NH_2_-PDI/TiO_2_/MoS_2_ formed ordered structures. Comparison of the XRD patterns in Fig. 3a,c,e,f confirms that the XRD patterns of the NH_2_-PDI/TiO_2_, MoS_2_/TiO_2_, and NH_2_-PDI/TiO_2_/MoS_2_ nanoparticles have good consistency with the XRD data of anatase phase TiO_2_ (JCPDS NO. 71–1167) at 2θ degrees of 25.3°, 37.7°, 48.0°, 53.8°, 55.0°, 62.6°, 58.6°, 70.6°, and 75.2°^36^. Compare with pure TiO_2,_ the crystallization properties of the two composites were slightly weakened, which should be due to the binding of NH_2_-PDI (Fig. 3c,f). Since the NH_2_-PDI/TiO_2_/MoS_2_ composite was loaded with very little NH_2_-PDI and MoS_2_, the characteristic peaks of NH_2_-PDI and MoS_2_ did not appear in its XRD patterns (Fig. 3f). Additionally, compared with pure TiO_2_, NH_2_-PDI/TiO_2_/MoS_2_ exhibited a widened peak width. This may be caused by the formation of heterojunction between TiO_2_, NH_2_-PDI and MoS_2_, and the heterojunction lead to lattice distortion of TiO_2_. The average particle sizes of TiO_2_, NH_2_-PDI/TiO_2_, MoS_2_/TiO_2_, and NH_2_-PDI/TiO_2_/MoS_2_ calculated by Scherrer’s equation were 10.2 nm, 12.5 nm, 11.3 nm, and 13.6 nm, respectively^37^.

Raman spectroscopy was applied to further check the phase and formation of the NH_2_-PDI/TiO_2_/MoS_2_ nanocomposite. As shown in Fig. S-3, the pristine TiO_2_ nanoparticles exhibit five peaks at 145.1 ± 0.2, 196.6 ± 0.1, 396.1 ± 0.2, 513.7 ± 0.2, and 638.1 ± 0.1 cm^−1^, which belong to the *E*_g_, *E*_g_, *B*_1g_, *A*_1g_, and *E*_g_ anatase tetragonal vibration modes of TiO_2_, respectively^38^. The *E*_g_, *B*_1g_, and *A*_1g_ peaks correspond to symmetric stretching, symmetric bending, and antisymmetric bending vibrations of O-Ti–O, respectively. The results demonstrate the existence of typical anatase TiO_2_ phase, and it is consistent with the XRD results. Characteristic peaks of MoS_2_ (A_1g_ and *E*^1^_2g_ modes) were observed in the Raman spectra of NH_2_-PDI/TiO_2_/MoS_2_ photocatalyst, indicating that MoS_2_ was loaded on the surface of TiO_2_^39^. Compared with pure TiO_2_, NH_2_-PDI/TiO_2_/MoS_2_ photocatalyst exhibited red shifts of *E*_g_, *E*_g_, *A*_1g_, and *E*_g_ modes (145.6 ± 0.3, 197.8 ± 0.1, 514.1 ± 0.1, and 638.4 ± 0.1 cm^−1^). This indicates that there are strong intimate interactions between TiO_2_, MoS_2_, and NH_2_-PDI.

**Figure 4:**
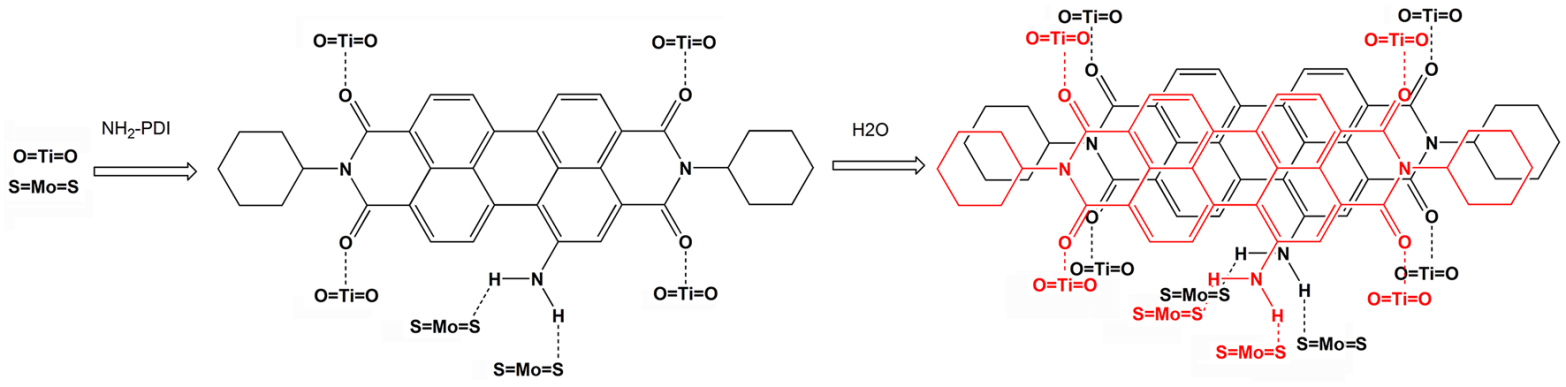
Graphical representation of the investigated adsorption mode of NH_2_-PDI onto the TiO_2_ and MoS_2_ surface.

FT-IR measurements revealed the connection between TiO_2_, NH_2_-PDI and MoS_2_ in NH_2_-PDI/TiO_2_/MoS_2_ nanocomposite (Fig. S-4). As shown in Fig. S-4a, for pure TiO_2_, the vibration modes observed at 617 cm^−1^ and 749 cm^−1^ are due to the O–Ti–O bending and Ti–O stretching vibrations, respectively. For MoS_2_, the vibration mode at 1389 cm^−1^ is assigned to Mo–S vibration, while the vibration mode at 1550 cm^−1^ may be S–O asymmetric stretching (Fig. S-4d)^40^. In MoS_2_/TiO_2_ and NH_2_-PDI/TiO_2_/MoS_2_, all characteristic vibration modes were present but with very low Mo–S and S–O vibration modes, which indicates that the MoS_2_/TiO_2_ and NH_2_-PDI/TiO_2_/MoS_2_ contain very little amount of MoS_2_ (Fig. S-4e, f). Remarkably, the bending vibration of C-H at 2926–2817 cm^−1^ observed in NH_2_-PDI shifted to 2968–2875 cm^−1^ in NH_2_-PDI/TiO_2_ and to 2992–2889 cm^−1^ in NH_2_-PDI/TiO_2_/MoS_2_, which may be an indicative of the binding between NH_2_-PDI and TiO_2_ or MoS_2_. In addition, the stretching vibration of C = C in NH_2_-PDI at 1645 cm^−1^ shifted to 1639 cm^−1^ in NH_2_-PDI/TiO_2_ and to 1627 cm^−1^ in NH_2_-PDI/TiO_2_/MoS_2_ nanocomposite. Compared with NH_2_-PDI, the C=C absorption peak of NH_2_-PDI/TiO_2_/MoS_2_ was blue shifted, indicating that the π-π interaction within NH_2_-PDI decreased. The absorption peak around 3300 cm^−1^ was caused by the stretching vibration of O–H bond, which was related to the atmospheric water adsorbed on the catalyst surface. As shown in Fig. 4, the Ti…O bond and S…H bond can be formed when NH_2_-PDI meets with TiO_2_ and MoS_2_. After the addition of deionized water, NH_2_-PDI self-assembled through intermolecular π–π stacking interactions. For NH_2_-PDI, dichloromethane is a “good” solvent, while water is a “poor” solvent. When the “poor” solvent was added, the solubility of originally dissolved NH_2_-PDI was limited, and NH_2_-PDI self-assembled through non-covalent interactions and precipitated into solid.

**Figure 5:**
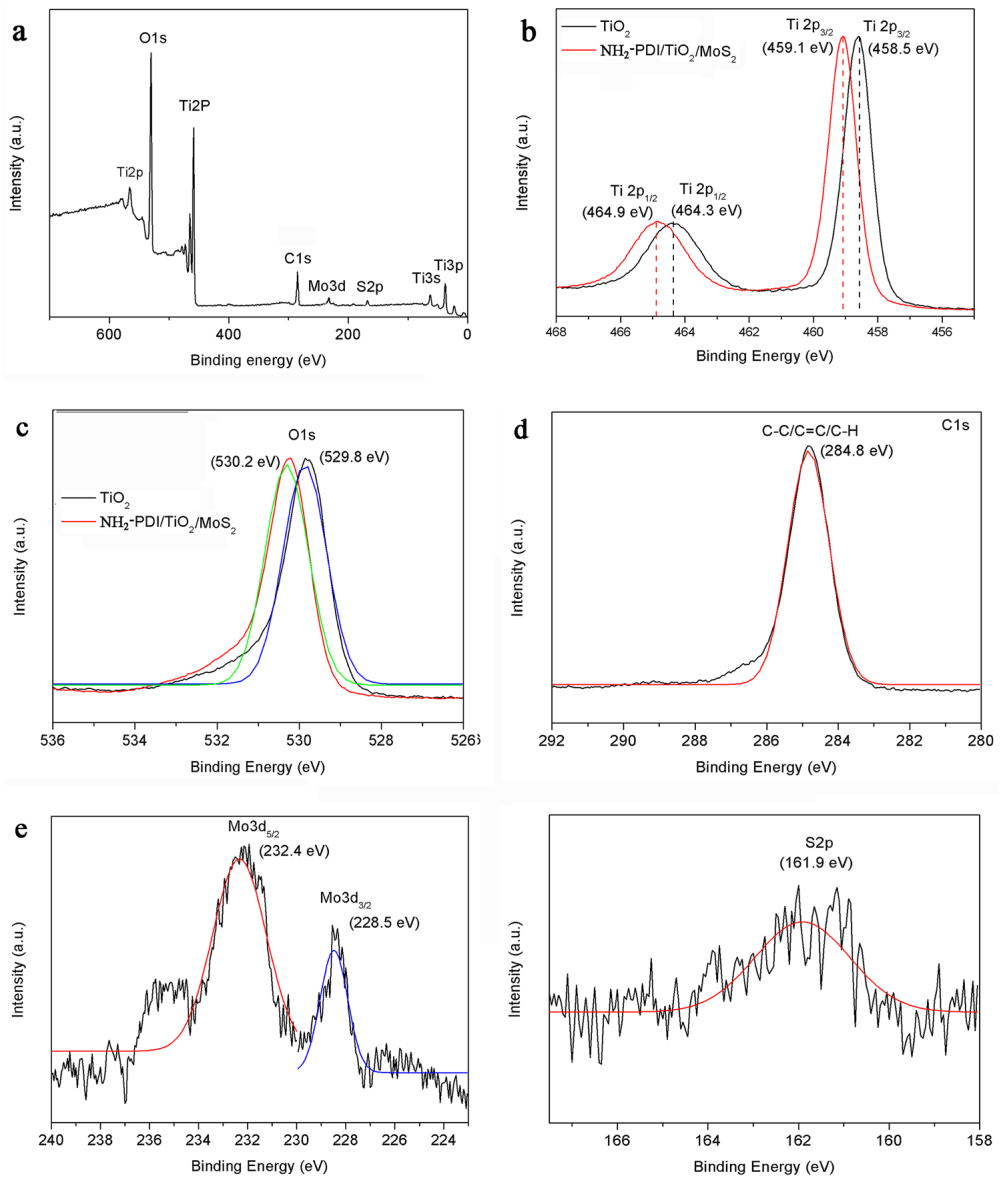
XPS spectra of (**a**) survey spectra in NH_2_-PDI/TiO_2_/MoS_2_, (**b**) Ti2p in pure TiO_2_ and NH_2_-PDI/TiO_2_/MoS_2_, (**c**) O1s in pure TiO_2_ and NH_2_-PDI/TiO_2_/MoS_2_, (**d**) C1s in NH_2_-PDI/TiO_2_/MoS_2_, (**e**) Mo3d in NH_2_-PDI/TiO_2_/MoS_2_, and (**f**) S2p in NH_2_-PDI/TiO_2_/MoS_2_.

The chemical composition and valence state of NH_2_-PDI/TiO_2_/MoS_2_ sample were studied by XPS (Fig. 5). According to Fig. 5a, NH_2_-PDI/TiO_2_/MoS_2_ composite contains Ti, O, C, Mo, and S elements. For NH_2_-PDI/TiO_2_/MoS_2_ nanocomposite, the positions of Ti2p1/2 and Ti2p3/2 were observed at 464.9 eV and 459.1 eV, respectively. While for pure TiO_2_ nanoparticles, Ti2p1/2 and Ti2p3/2 peaks were observed at 464.3 eV and 458.5 eV, respectively (Fig. 5b)^41^. The O1s peak positions of NH_2_-PDI/TiO_2_/MoS_2_ and pure TiO_2_ were observed at 530.2 eV and 529.8 eV, respectively (Fig. 5c). These results indicate that after adding NH_2_-PDI and MoS_2_ to TiO_2_ nanoparticles, Ti2p peaks move to a higher energy by 0.6 eV than pure TiO_2_. Also, the peak position of O1s moved 0.4 eV towards high energy. The shifted spectrum implies the presence of more defects or adsorbed hydroxyl groups on the surface of NH_2_-PDI/TiO_2_/MoS_2_^42^. These defect states may serve as shallow donors to enhance charge transfer at the multiple interfaces and thus improve the overall degradation efficiency towards dyes. In NH_2_-PDI/TiO_2_/MoS_2_ sample, The C1s spectra (Fig. 5d) shows the corresponding peak at 284.8 eV, which can be identified as C–C/C=C/C–H functional groups. The positions of the Mo3d5/2 and Mo3d3/2 peaks were observed at 232.4 eV and 228.5 eV, respectively. Similarly, the position of S2p was observed at 161.9 eV^43^.

The surface areas of TiO_2_, NH_2_-PDI, NH_2_-PDI/TiO_2_, MoS_2_, MoS_2_/TiO_2_, and NH_2_-PDI/TiO_2_/MoS_2_ were determined by nitrogen adsorption method. The BET surface area of NH_2_-PDI/TiO_2_/MoS_2_ (39 m^2^/g) is higher than that of TiO_2_ (21 m^2^/g), NH_2_-PDI/TiO_2_ (37 m^2^/g), MoS_2_ (23 m^2^/g), or MoS_2_/TiO_2_ (26 m^2^/g). Therefore, it can be speculated that the large surface area of NH_2_-PDI/TiO_2_/MoS_2_ could promote the photocatalytic activity.

**Figure 6:**
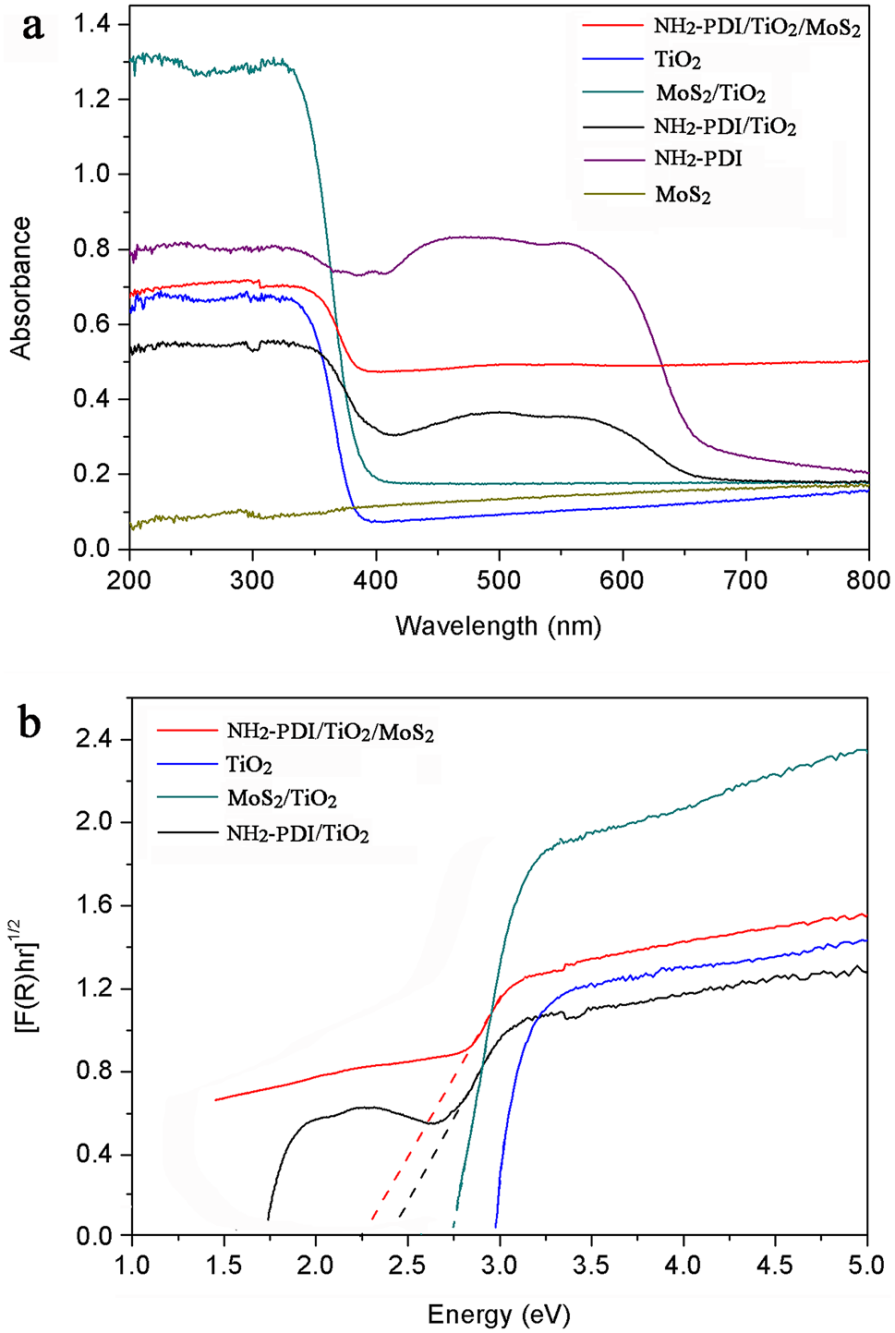
(**a**) UV–vis diffuse reflection spectra (DRS) and (**b**) The corresponding Tauc plot of samples.

The UV–vis diffuse reflection spectra (DRS) and the corresponding Tauc plots of samples are shown in Fig. 6. Pure TiO_2_ absorbs only UV light and exhibits absorption below 380 nm. Adding NH_2_-PDI and MoS_2_ nanoflakes to TiO_2_ nanoparticles can result in more light-harvesting in visible region (Fig. 6a). The absorption intensity of NH_2_-PDI/TiO_2_/MoS_2_ in visible light region was obviously enhanced, which could be attributed to the visible light absorption characteristics of NH_2_-PDI and MoS_2_ nanoflakes. The band gap energy of the samples can be estimated by the formula Eg (eV) = 1240/λ_AE_ (nm) using the position of the absorption edge (λ_AE_)^44^. The downward slopes of the absorption curves are extrapolated to cross the X-axis and the λ_AE_-values of TiO_2_, MoS_2_/TiO_2_, NH_2_-PDI/TiO_2_, and NH_2_-PDI/TiO_2_/MoS_2_ samples were estimated to be at 389 nm, 423 nm, 459 nm, and 467 nm, respectively (Fig. S-5). The Eg values of TiO_2_, MoS_2_/TiO_2_, NH_2_-PDI/TiO_2_, and NH_2_-PDI/TiO_2_/MoS_2_ samples were estimated to be at 3.18, 2.93, 2.70, and 2.66 eV, respectively.

The band gaps (Eg, eV) of the samples are also calculated by the Tauc equation and Kubelka–Munk function^45^:

1 $${[F(R)h\upsilon]^0.5} =A(h\upsilon-Eg)$$

2 $$F(R)={(1-R)^{2}}/2R$$

where, R is the calibrated reflection of samples with BaSO_4_ reflection, and F(R) is proportional to the absorption constant. h and ν represent the Planck constant and frequency, while A and Eg represent constant and band gap energy, respectively. The [F(R)hν]^0.5^ versus hν is shown in Fig. 6b. Extrapolation of the linear portion at [F(R)hν]^0.5^ = 0 provides Eg value of the samples. The band gaps were found to be 2.98, 2.75, 2.47, and 2.27 eV for TiO_2_, MoS_2_/TiO_2_, NH_2_-PDI/TiO_2_, and NH_2_-PDI/TiO_2_/MoS_2_, respectively. The increase of UV–visible light absorption and decrease of band gap energy enhanced the photodegradation efficiency of NH_2_-PDI/TiO_2_/MoS_2_ towards MB.

**Figure 7:**
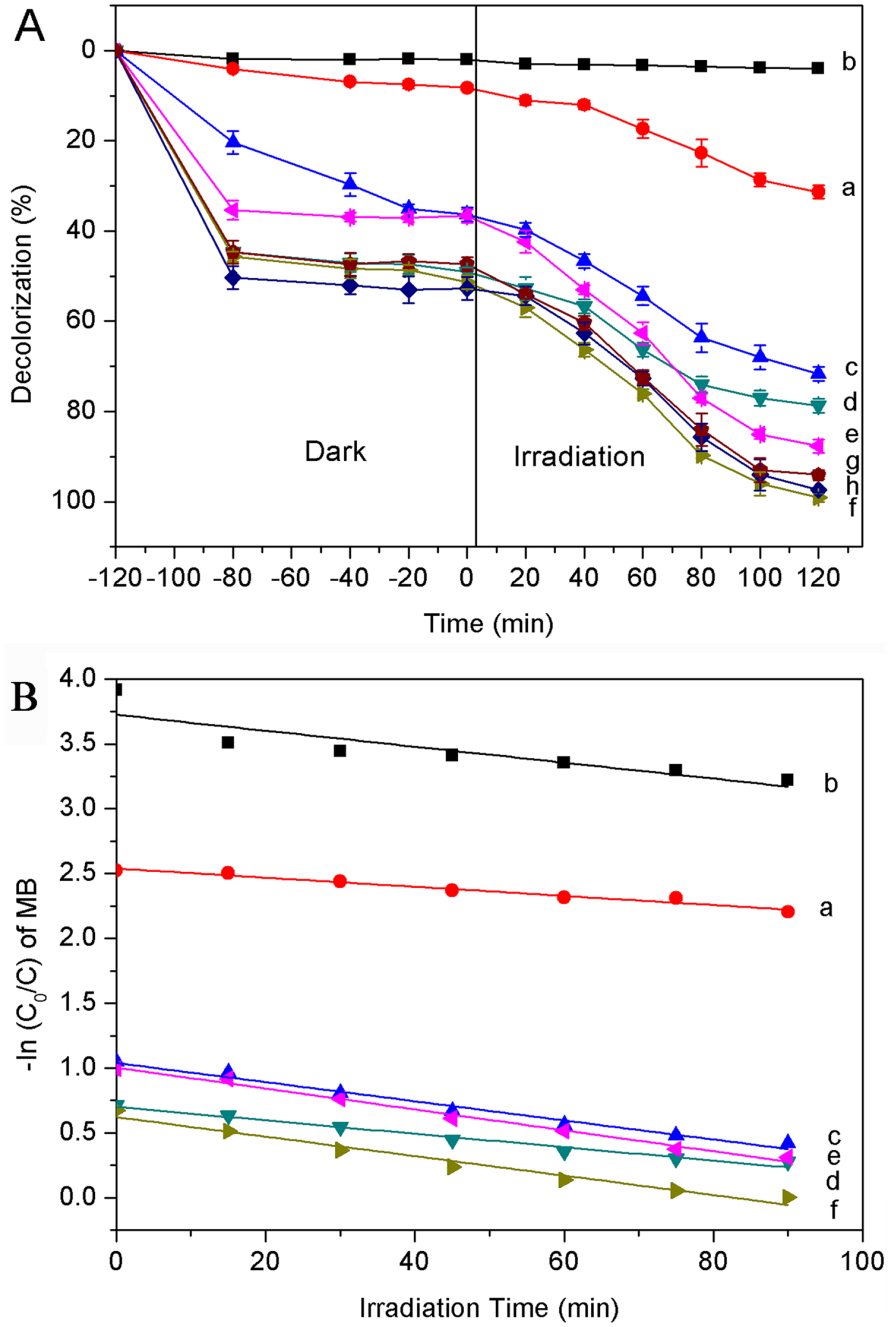
(**A**) The activity diagram of MB degradation by different catalysts in visible light, (**B**) The first order kinetics curve fitting of MB degradation by different catalysts. (a) TiO_2_, (b) NH_2_-PDI, (c) NH_2_-PDI/TiO_2_, (d) MoS_2_, (e) MoS_2_/TiO_2_, (f) NH_2_-PDI/TiO_2_/2%MoS_2_, (g) NH_2_-PDI/TiO_2_/1%MoS_2_, and (h) NH_2_-PDI/TiO_2_/3%MoS_2_. [MB] = 0.01 g/L, pH = 7, catalyst suspended = 1 g/L.

The activity diagram of MB degradation by different catalysts in visible light and the first order kinetics curve fitting of MB degradation by different catalysts are shown in Fig. 7. Prior to the illumination, each catalyst was dispersed in the dye solution. After 120 min of vigorous magnetic stirring under dark condition, 51.3 ± 1.5% of MB was absorbed by NH_2_-PDI/TiO_2_/MoS_2_, whereas pure TiO_2_ only absorbed 8.2 ± 0.7% of MB. It can be seen that the spongy NH_2_-PDI clusters and multi-layer MoS_2_ nanoflowers decorated TiO_2_ exhibited extremely high adsorption efficiency under dark condition. Both MoS_2_ and TiO_2_/MoS_2_ have strong adsorption capacity, which may be due to the flower-like MoS_2_ with negative surface charge promoted the adsorption of cationic dye MB. It can be noted that after 120 min of irradiation, the degradation rate of dye was 99.0 ± 1.0% for NH_2_-PDI/TiO_2_/MoS_2_, while the corresponding data was 4.0 ± 0.3%, 31.3 ± 1.5%, 71.7 ± 1.6%, 78.7 ± 1.3%, and 87.6 ± 1.5% for NH_2_-PDI, TiO_2_, NH_2_-PDI/TiO_2_, MoS_2_, and MoS_2_/TiO_2_, respectively (Fig. 7A). Though TiO_2_ has no visible light absorption, it has been found to be solar active. This may be due to the dye sensitization mechanism of MB to TiO_2_. For better comparison, NH_2_-PDI/TiO_2_/1%MoS_2_ or NH_2_-PDI/TiO_2_/3%MoS_2_ photocatalysts was used for dye degradation under identical conditions. As a result, 97.3 ± 0.6% or 94.0 ± 1.1% of dye were degraded respectively under visible light irradiation in 120 min. The results show that 0.1%NH_2_-PDI/TiO_2_/2%MoS_2_ had higher efficiency in MB degradation than other catalysts. The kinetics plot shows that ln (C_0_/C) has linear relationships with reaction time, indicating that the photodegradation of MB follows first-order kinetics (Fig. 7B). The apparent rate constants (k) were determined as 0.00616, 0.00352, 0.00738, 0.00517, 0.00752, and 0.00806 min^−1^ for TiO_2_, NH_2_-PDI, NH_2_-PDI/TiO_2_, MoS_2_, MoS_2_/TiO_2_, and NH_2_-PDI/TiO_2_/MoS_2_, respectively. These results confirmed the high photocatalytic activity of NH_2_-PDI/TiO_2_/MoS_2_. Fig. S-6 shows the decolorization of MB dye (0.01 g/L) within 90 min in the presence of NH_2_-PDI/TiO_2_/MoS_2_ catalyst. The decrease of absorption spectrum indicates decolorization of the dye under the applied conditions. There were no additional peaks in the UV–Vis spectrum, indicating that the dye was completely degraded. It is reported that the products of the decolorization process are H_2_O, CO_2_, NO_2_, and SO_2_^46^, and the mechanism of MB degradation is shown in Fig. S-7.

Chemical oxygen demand (COD) is an indicator that reflecting the required oxygen for the oxidation of organic matters present in solution^47^. A smaller COD value means a lower pollution of the sample. The mineralization degree of MB was calculated by COD result, and the kinetic results shown that the k value of NH_2_-PDI/TiO_2_/MoS_2_ was 0.0076 min^−1^ (Fig. S-8). The value correspond to t_1/2_ values of 85.9 and 91.2 min (based on t_1/2_ = 0.693/k) for the degradation and mineralization of MB molecules, respectively^48^. These confirmed that the photodegradation of MB was 1.06 times faster than its mineralization. The results confirmed a relatively smaller mineralization extent for MB molecules than its photodegradation extent.

The photonic efficiency of MB dye degradation by NH_2_-PDI/TiO_2_/MoS_2_ under optimum condition was calculated using a reported method^49^. The quantum yield of reaction (Ø) is defined as follows (Eq. (1)):

$$\O =\frac{\mathrm{Number \,of\, molecules\, decomposed }}{\mathrm{Number\, of\, photons\, of\, light\, absorbed}}$$

The photo-degradation rate constants (*k*’) of MB dyes under monochromatic light source can also be used to calculate the reaction quantum yield (Eq. (2)).

$$\O =\frac{k^{\prime}}{303I\varepsilon l}$$

where Ø is the reaction of quantum yield. *I* is the light intensity range in 400–800 nm (2.312 × 10^–3^ Einstein). *ɛ* is the molar absorptivity of MB at 664 nm (25,440 cm^–1^ M^–1^). *l* is the path length of reaction tube, which is 0.23 m for 50 mL solution. The degradation quantum yields obtained with TiO_2_, NH_2_-PDI, NH_2_-PDI/TiO_2_, MoS_2_, MoS_2_/TiO_2_, and NH_2_-PDI/TiO_2_/MoS_2_ were 0.000152, 0.000869, 0.000182, 0.000128 and 0.000186, respectively. These results indicate that the quantum yield of NH_2_-PDI/TiO_2_/MoS_2_ catalyzes process was higher than that of other processes.

During the process of MB degradation, a large amount of MB was adsorbed on the surface of catalysts. The visible light driven catalysis of the NH_2_-PDI/TiO_2_/MoS_2_ may be enhanced due to the effect of dye sensitization. Therefore, amoxicillin (AMX, a colorless medical antibiotic) was selected to evaluate the photocatalytic activity of NH_2_-PDI/TiO_2_/MoS_2_ nanoparticles under the same conditions. The photocatalytic degradation results of AMX are shown in Fig. S-9. NH_2_-PDI/TiO_2_/MoS_2_ exhibited higher visible photocatalytic activity than NH_2_-PDI/TiO_2_ or MoS_2_/TiO_2_. When AMX solution was mixed with NH_2_-PDI/TiO_2_/MoS_2_ in the absence of light, the removal rate of AMX was 48%. When NH_2_-PDI/TiO_2_ or MoS_2_/TiO_2_ was used under visible light irradiation for 120 min, the degradation rates of AMX were 56.9% or 71.2%, respectively. Comparatively, NH_2_-PDI/TiO_2_/MoS_2_ exhibited superior photodegradation performance (84.8%). The degradation processes conforms to pseudo-first-order kinetics. The photocatalytic activity of NH_2_-PDI/TiO_2_/MoS_2_ reached a maximum value of 0.00986 min^−1^, which was about 1.67 times and 1.79 times higher than NH_2_-PDI/TiO_2_ and MoS_2_/TiO_2_ (0.00590 min^−1^ and 0.00551 min^−1^), respectively. The result indicates that NH_2_-PDI/TiO_2_/MoS_2_ has strong visible-light driven photocatalytic activity.

The reusability and stability of a composite photocatalyst are important factor affecting its application (Fig. S-10). The NH_2_-PDI/TiO_2_/MoS_2_ was used for the degradation of different solutions of MB for six consecutive experiments under the same reaction conditions. After each experiment, the catalyst was recovered by centrifugation and dried in an air oven. The degradation efficiency of NH_2_-PDI/TiO_2_/MoS_2_ reduced from 99 ± 0.9% to 89 ± 2.3% after six cycles of reuse, which was probably due to a small loss of catalyst during recycle (Fig. S-10a). NH_2_-PDI/TiO_2_/MoS_2_ was prepared by hydrothermal synthesis method, which made the contact between three monomers more compacted and reduced the dissolution of MoS_2_ or NH_2_-PDI molecule. Therefore, the composite catalyst exhibits high stability in the degradation process. In addition, the composite material packaged TiO_2_ by NH_2_-PDI and MoS_2_. The contact area between the three semiconductors increases due to the point-to-surface contact. Due to the close contact interface, the interactions between the three semiconductors would also be more intense. The π-conjugated electrons in NH_2_-PDI can be quickly transferred from the interior of the semiconductor to the surface, and then to the surface of TiO_2_ or MoS_2_. This reduces electrons and holes recombination and improves the photocatalytic activity. Fig. S-10b shows the FT-IR spectra of the NH_2_-PDI/TiO_2_/MoS_2_ photocatalyst before and after six cycles of photocatalytic degradation of MB. It can be seen that the spectrum of the regenerated photocatalyst was basically the same as that of the fresh photocatalyst. There is no spectrum of MB, which proves that MB has been completely degraded rather than adsorbed on the catalyst surface during the illumination process. The result of UV–vis spectra shows that the concentration of organic matter in the washing solution of catalyst was very low. Few intermediate species and NH_2_-PDI were detected, indicating that the dissolution of NH_2_-PDI in the composite structure was very few and MB was mineralized to CO_2_. These results show that the NH_2_-PDI/TiO_2_/MoS_2_ catalyst is stable and reusable.

The photoluminescence (PL) spectrum was used to analyze the recombination of electron hole pairs. In general, a higher peak indicates a more rapid charge recombination. As shown in Fig. S-11, the peak at 399 nm is attributed to a direct transition from the conduction band to the valence band. Moreover, the luminescence peaks at 440, 451, and 469 nm are caused by inter-band transitions, and the peaks at 483 and 492 nm are caused by intra-band transitions within the energy level traps or surface states^50^. The peak intensity was arranged as follow: TiO_2_ > NH_2_-PDI/TiO_2_ > MoS_2_/TiO_2_ > NH_2_-PDI/TiO_2_/MoS_2_. Especially, the peak intensity of NH_2_-PDI/TiO_2_/MoS_2_ composite was much weaker than that of the pure TiO_2_. The –C=O…H hydrogen bond between the -OH of TiO_2_ and –C=O of NH_2_-PDI, and the S…H bond between the -S of MoS_2_ and -NH_2_ of NH_2_-PDI acted as short and fast channels for migrating the photogenerated charge carriers from NH_2_-PDI to TiO_2_ and MoS_2_. Based on the above results, the introduction of NH_2_-PDI and MoS_2_ can greatly accelerate the charge transfer process.

**Figure 8:**
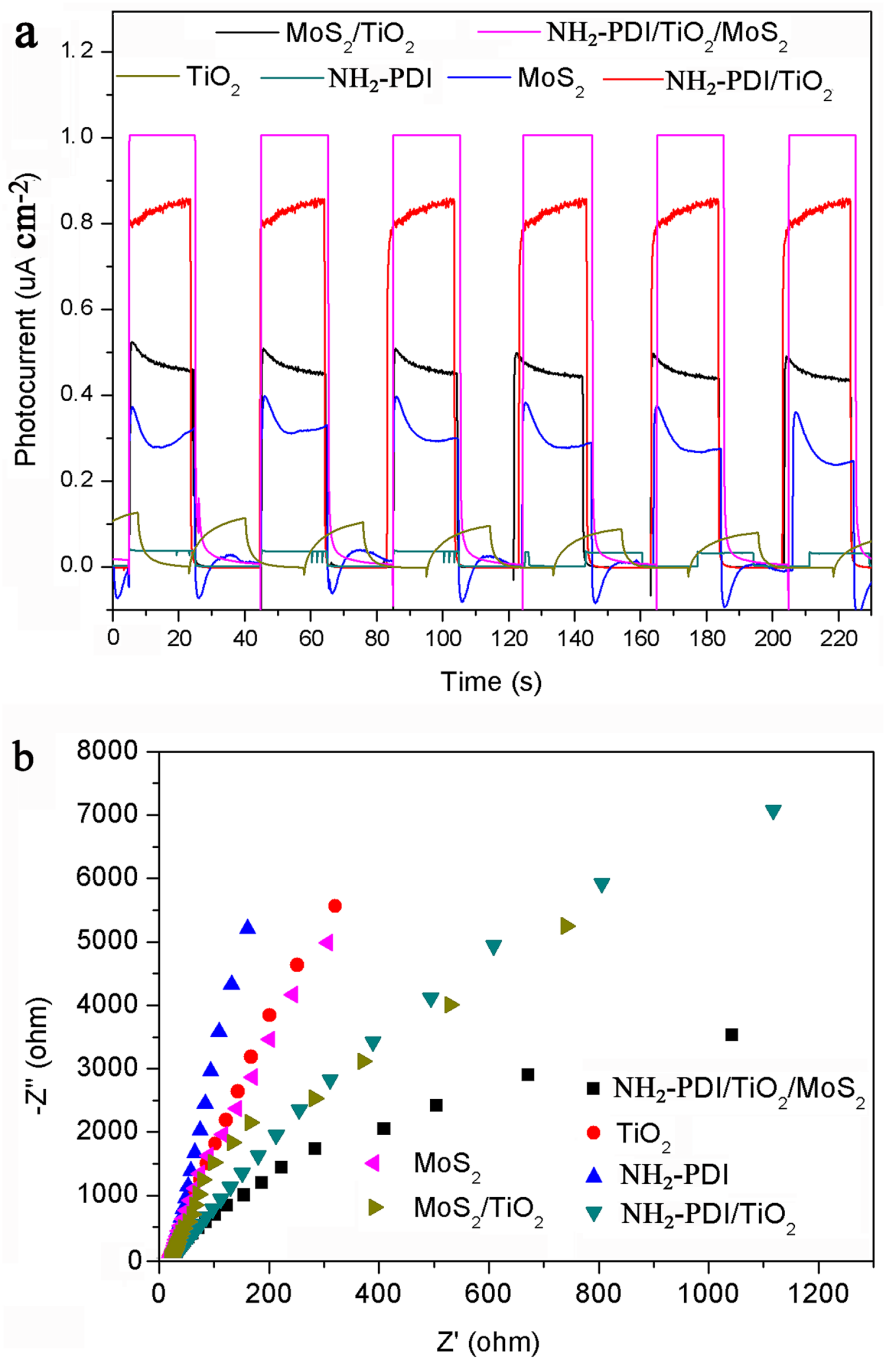
(**a**) Photocurrent transient responses and (**b**) EIS spectra of TiO_2_, NH_2_-PDI, NH_2_-PDI/TiO_2_, MoS_2_, MoS_2_/TiO_2_, and NH_2_-PDI/TiO_2_/MoS_2_ at a constant potential of 0.4 V with simulated solar light at 300 mW/cm^2^. frequency: 0–10,000 Hz, electrolyte: 0.1 M Na_2_SO_4_, amplitude: 10 mV.

The mechanism of enhanced photocatalytic activity of NH_2_-PDI/TiO_2_/MoS_2_ composite was further studied by photoelectrochemistry method. The separation of photogenerated electrons and holes plays an important role in the photocatalytic decomposition of organic pollutants, which can be evaluated by transient photocurrent responses (*I-t*) and electrochemical impedance spectra (*EIS*) (Fig. 8). Figure 8a shows the *I-t* curves of TiO_2_, NH_2_-PDI, NH_2_-PDI/TiO_2_, MoS_2_, MoS_2_/TiO_2_, and NH_2_-PDI/TiO_2_/MoS_2_ under several intermittent visible light irradiation cycles. It can be seen that the photocurrent of all samples exhibited good repeatability when the light was turned on and off. The light current response of pure TiO_2_ and NH_2_-PDI were very weak (only 0.119 and 0.035 μA cm^−2^, respectively). NH_2_-PDI/TiO_2_/MoS_2_ composite had the largest photocurrent response, and the stable photocurrent was about 1.007 μA cm^-2^, which was higher than that of MoS_2_ (0.372 μA cm^−2^), MoS_2_/TiO_2_ (0.522 μA cm^−2^), and NH_2_-PDI/TiO_2_ (0.846 μA cm^−2^). The enhanced photocurrent response indicates that the separation efficiency of photogenerated carriers and the photocatalytic performance were improved. Figure 8b displays the EIS Nyquist plots of as-prepared samples. The radius of the arc on the EIS spectrum reflects the surface charge transfer resistance and the solid interface delamination resistance. The smaller the semicircle arc is, the easier the charge transfer proceeds^51^. Well discussion on EIS spectra and the resulted Bodes' plots has been presented in previous work^52^. Here, the following trend was obtained for the arc radius of the Nyquist plots, confirming that the NH_2_-PDI/TiO_2_/MoS_2_ composite have better charge transfer capability.

NH_2_-PDI > TiO_2_ > MoS_2_ > NH_2_-PDI/TiO_2_ > MoS_2_/TiO_2_ > NH_2_-PDI/TiO_2_/MoS_2_.

The Mott-Schottky plots were used to estimate the conduction band (CB) of the TiO_2_, NH_2_-PDI, and MoS_2_, which were − 1.26 eV, − 1.61 eV, and − 0.75 eV, respectively (Fig. S-12). Combining with their bandgaps (TiO_2_ at 2.98 eV [Fig. 5b], NH_2_-PDI at 2.36 eV^30^, and MoS_2_ at 1.75 eV^26^), the CB/VB (conduction band/valence band) of TiO_2_, NH_2_-PDI, and MoS_2_ were evaluated to be − 1.26/1.72 eV, − 1.56/0.8 eV, and − 0.75/1.00 eV, respectively. It can be found that the CB position of singlet oxygen (·O_2_^-^) (− 0.18 eV) is shallower than that of NH_2_-PDI, TiO_2_, and MoS_2_. This result indicates that ·O_2_^-^ may be the main active substance in NH_2_-PDI/TiO_2_/MoS_2_ composite for photocatalytic degradation of MB. The VB positions of MoS_2_ was shallower than that of hydroxyl radical (·OH) (2.40 eV)^53^, so it is possible that the ·OH cannot be generated by NH_2_-PDI/TiO_2_/MoS_2_.

The active species in the photocatalytic process was studied by free radical capture experiment^53^. As can be seen from Fig. S-13, with the addition of p-BQ scavenger, the degradation rate decreased from 99.9% to 33%, indicating that ·O_2_^−^ is one of the main active species in the photocatalytic degradation process. In the presence of EDTA-2Na, the degradation efficiency was significantly reduced, indicating that h^+^ is also one of the major active radicals. After the addition of IPA, the degradation activity of the catalyst did not decrease, indicating that ·OH is not the main active species in MB degradation.

**Figure 9:**
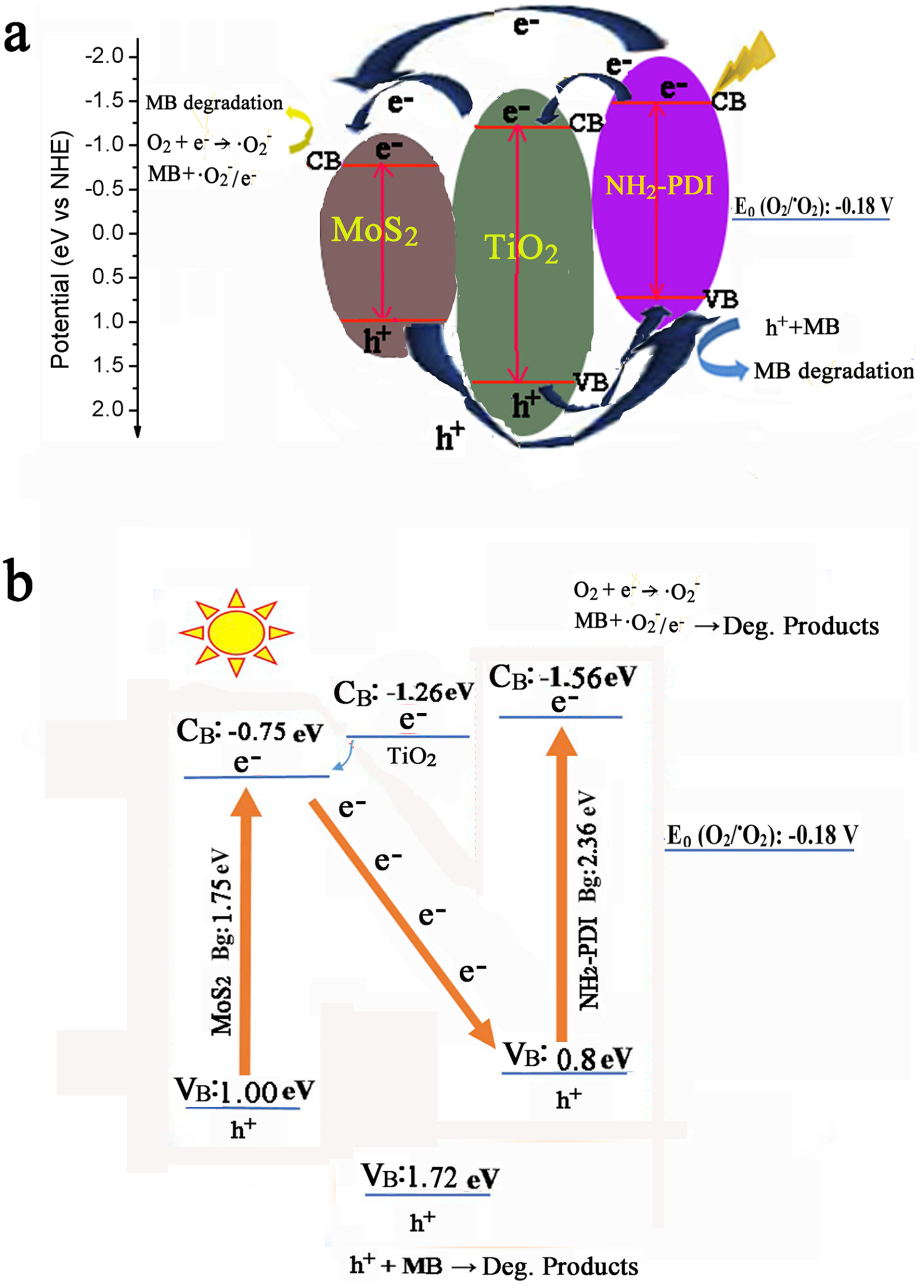
Schematic illustration of the photocatalytic mechanism over the NH_2_-PDI/TiO_2_/MoS_2_ heterostructure. (**a**) Type II-Heterojunction and (**b**) Z-scheme pathways.

Based on the results above, two mechanisms including ‘type II- heterojunction’ and ‘Z-scheme’ were suggested to illustrate the photodegradation pathways occurred in this work (Fig. 9)^54^. In ‘type II- heterojunction’ mechanism (Fig. 9a), both the NH_2_-PDI and MoS_2_ semiconductors can be excited by the arrived photons under visible light irradiation, resulting in production of e^-^/h^+^ pairs in both semiconductors. As the CB and VB of NH_2_-PDI are located at higher energy levels than those of TiO_2_ and MoS_2_, the photogenerated electrons in the CB of NH_2_-PDI can transfer to the CB of TiO_2_, and then to the CB of MoS_2_, while holes travel in the opposite direction in the VB. These charge carriers’ transfer processes result in the accumulation of the photogenerated electron in the CB level of MoS_2_ and the photogenerated holes in the VB level of NH_2_-PDI. While in the ‘Z-scheme’ mechanism (Fig. 9b), the photogenerated electrons at the CB level of MoS_2_ can migrate to the VB level of NH_2_-PDI based on the standard potentials, and thus, the ‘Z-scheme’ pathway accumulates the photogenerated electrons at the CB level of NH_2_-PDI and the photogenerated holes at the VB level of MoS_2_. The accumulated electrons at the CB level of NH_2_-PDI have the potential of − 1.61 eV, which is more negative than the potential of MoS_2_ (-0.75 eV). Therefore, the accumulated electrons at the CB level of NH_2_-PDI can effectively reduce dissolved oxygen and generate enough ·O_2_^−^ as an efficient reactive species for the degradation of MB. These holes are also stronger oxidizing agents than the accumulated holes in the VB level of NH_2_-PDI (accumulated in type II mechanism) that can directly oxidize MB molecules. Therefore, the ‘Z-scheme’ mechanism is a better illustration for the enhanced photocatalytic activity of NH_2_-PDI/TiO_2_/MoS_2_ composite in MB degradation than the ‘type II-heterojunction’ mechanism. Nevertheless, in both mechanisms, TiO_2_ facilitates the electron transfer between the conduction bands, reduces photoinduced charge carrier recombination and increases photocatalytic activity.

## Conclusions

In conclusion, NH_2_-PDI/TiO_2_/MoS_2_ ternary composite was successfully synthesized by hydrothermal synthesis method. Morphological and chemical features of TiO_2_, NH_2_-PDI, NH_2_-PDI/TiO_2_, MoS_2_, MoS_2_/TiO_2_, and NH_2_-PDI/TiO_2_/MoS_2_ composites were examined by SEM, XRD, FTIR, XPS, and DRS. SEM results demonstrate that NH_2_-PDI nanorods and MoS_2_ nanoflowers mixed with TiO_2_, which can provide more active sites and mass charge transport pathways in the catalytic system. The photoelectrochemical measurement results show that the photocurrent performance of the ternary composite catalyst was superior to that of binary composites or pure NH_2_-PDI, MoS_2_, or TiO_2_. The NH_2_-PDI/TiO_2_/MoS_2_ exhibits excellent photocatalytic activity and high stability. The ‘Z-scheme’ accumulates more electrons at the CB level of NH_2_-PDI and photogenerated holes at the VB level of MoS_2_. The accumulated electrons can effectively reduce dissolved oxygen and generate enough ·O_2_^−^ as an efficient reactive species for the degradation of MB. These holes are also strong oxidizing agents that can directly oxidize MB molecules. TiO_2_ could reduce photoinduced charge carrier recombination and increases photocatalytic activity. This study highlights the potential application of organic and inorganic photocatalysts, and provides a feasible strategy for the photodegradation of dyes.

## Supplementary Information

Supplementary Information 1.
