# Supplementary material for: Hydrothermal synthesis of novel 1-aminoperylene diimide/TiO2/MoS2 composite with enhanced photocatalytic activity
Source: Sci Rep. 2020 Dec 15;10:22005. doi: 10.1038/s41598-020-78894-y (PMC7738548; doi:10.1038/s41598-020-78894-y)

**Supporting Information**

**Hydrothermal synthesis of novel 1-aminoperylene diimide/TiO_2_/MoS_2_ composite with enhanced photocatalytic activity**

Yongshan Ma ^a*^, Yue Wang ^b^, Tianyi Jiang ^a^, Fengxia Zhang ^a*^, Xuemei Li ^a^, Yanyan Zhu ^a^

*^a^ School of Municipal and Environmental Engineering, Shandong Jianzhu University, Jinan 250101, Shandong, China.*

*^b^ Shandong Provincial Key Laboratory of Metrology and Measurement, Shandong Institute of Metrology, Shandong Social Justice Institute of Metrology, Jinan 250014, P. R. China*

*^*^ Corresponding author. E-mail addresses: mlosh@sdjzu.edu.cn (Y. Ma), zhangfengxia19@sdjzu.edu.cn (F. Zhang).*


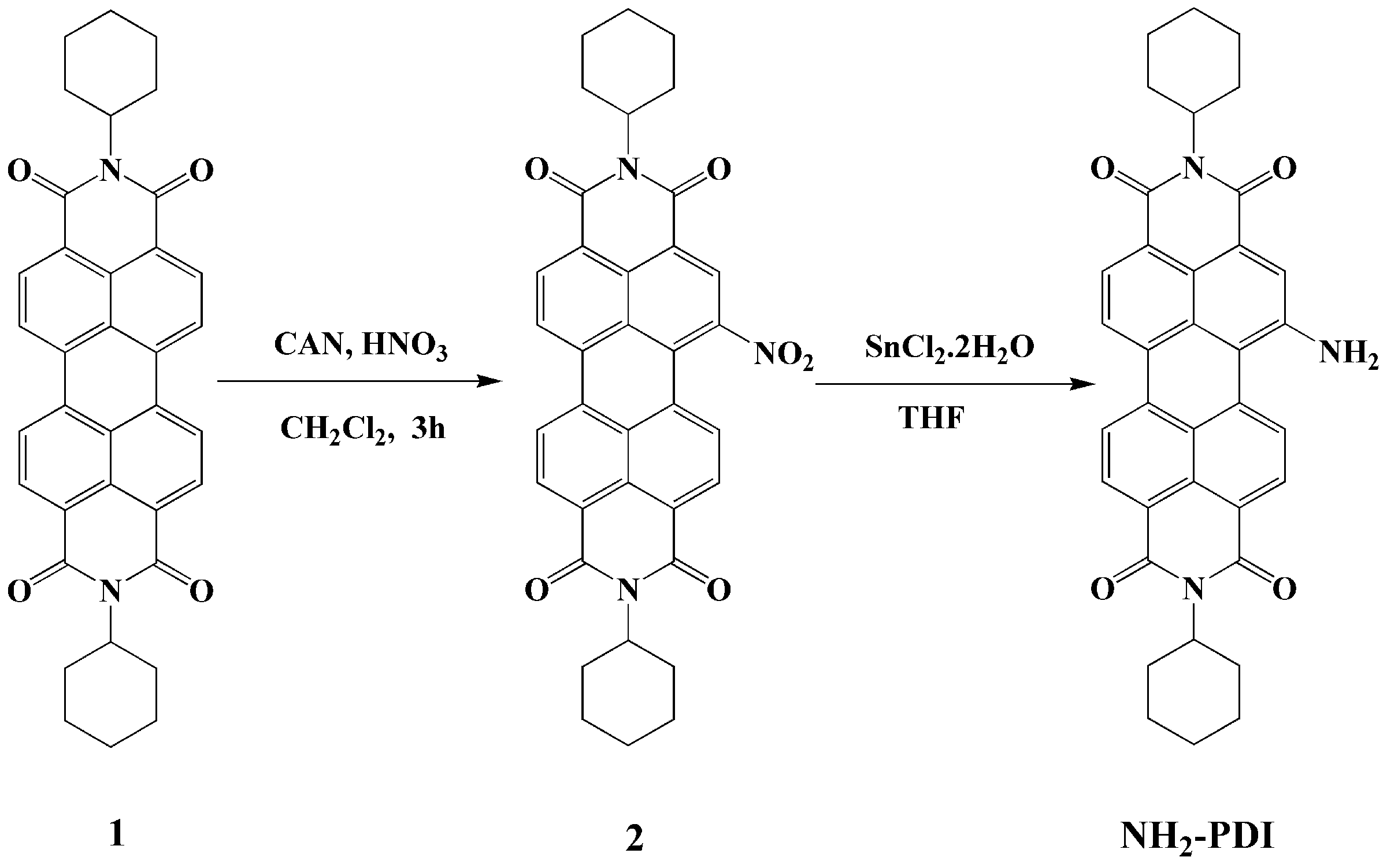
 Fig.S-1 Synthesis route of NH_2_-PDI.

**General procedure for the synthesis of NH_2_-PDI**

Compound 1 (2.0 g, 3.6 mmol), nitric acid (4.0 g, 63.4 mmol), cerium (IV) ammonium nitrate (CAN) (2.4 g, 4.4 mmol), and dichloromethane (200 mL) were stirred at 25 ℃ under argon atmosphere for 3 h. The mixture was neutralized with 10% KOH and extracted with dichloromethane. After solvent was removed, the crude product was purified by silica gel column chromatography with eluent dichloromethane to afford compound 2 (2.1g) in 95% yield. Then compound 2 (2.0 g, 3.4 mmol) and tin chloride dihydrate (10.0 g, 44 mmol) reacted for 3 h in THF at 80 ℃ under argon atmosphere to yield NH_2_-PDI in 69%. ^1^H-NMR(CDCl_3_, 300 MHz, ppm): δ=8.77 (d, J=8.4 Hz, 1H), 8.55-8.57 (m, 2H), 8.39-8.40 (m, 2H), 8.35 (d, J=8.0 Hz, 1H), 8.07 (s, 1H), 5.15 (s, 2H), 4.34-4.37 (m, 2H), 1.73-1.79 (m, 12H), 1.22-1.28 (m, 8H). HRMS: C_36_H_31_N_3_O_4_ (M^+^-H), calcd, 569.2393; found 569.2397.

Fig. S-2 EDX spectrum of NH_2_-PDI/TiO_2_/MoS_2_ photocatalyst.


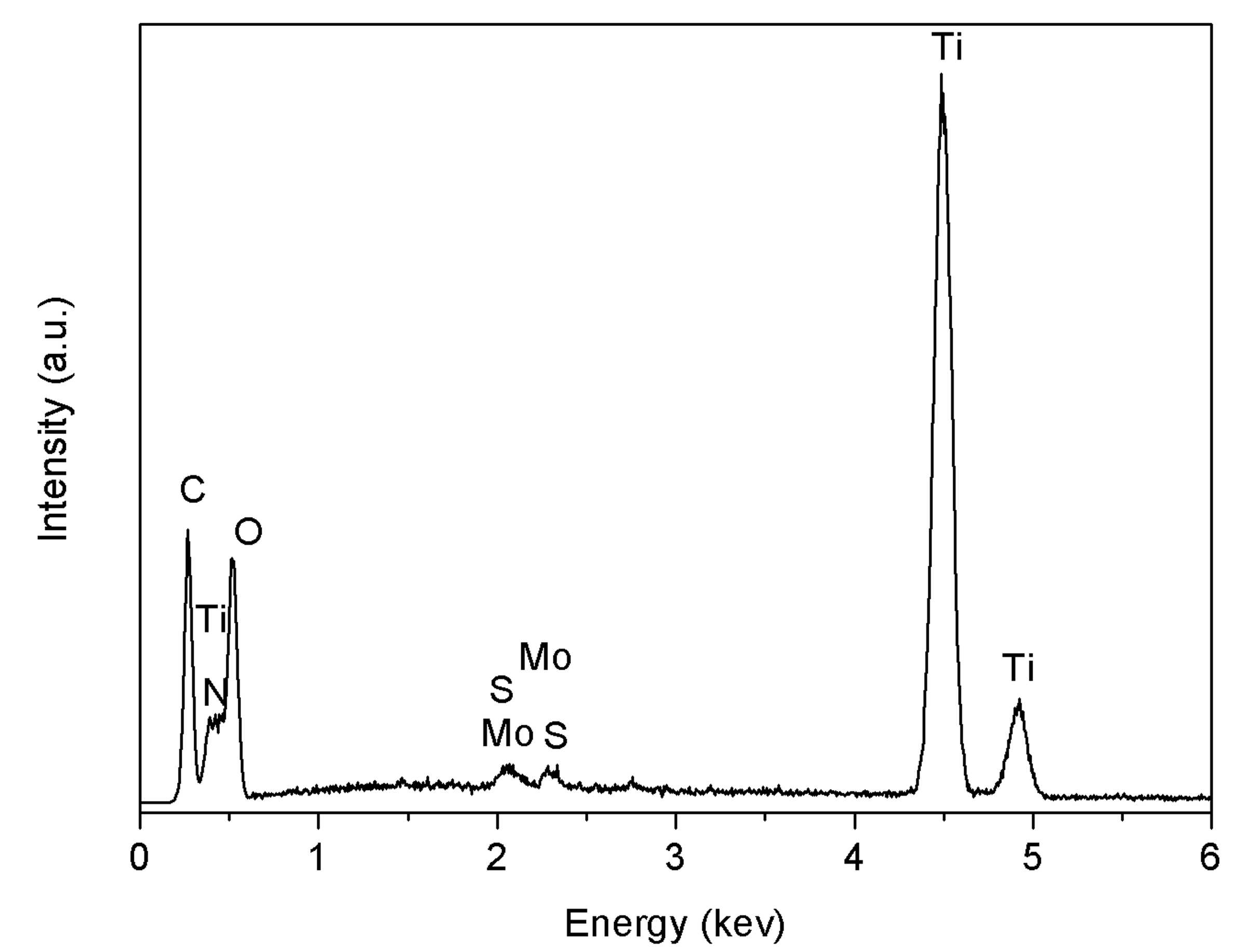


Fig. S-3 Raman spectra of (a) pristine TiO_2_ (b) NH_2_-PDI/TiO_2_/MoS_2_ nanocomposite samples.

**
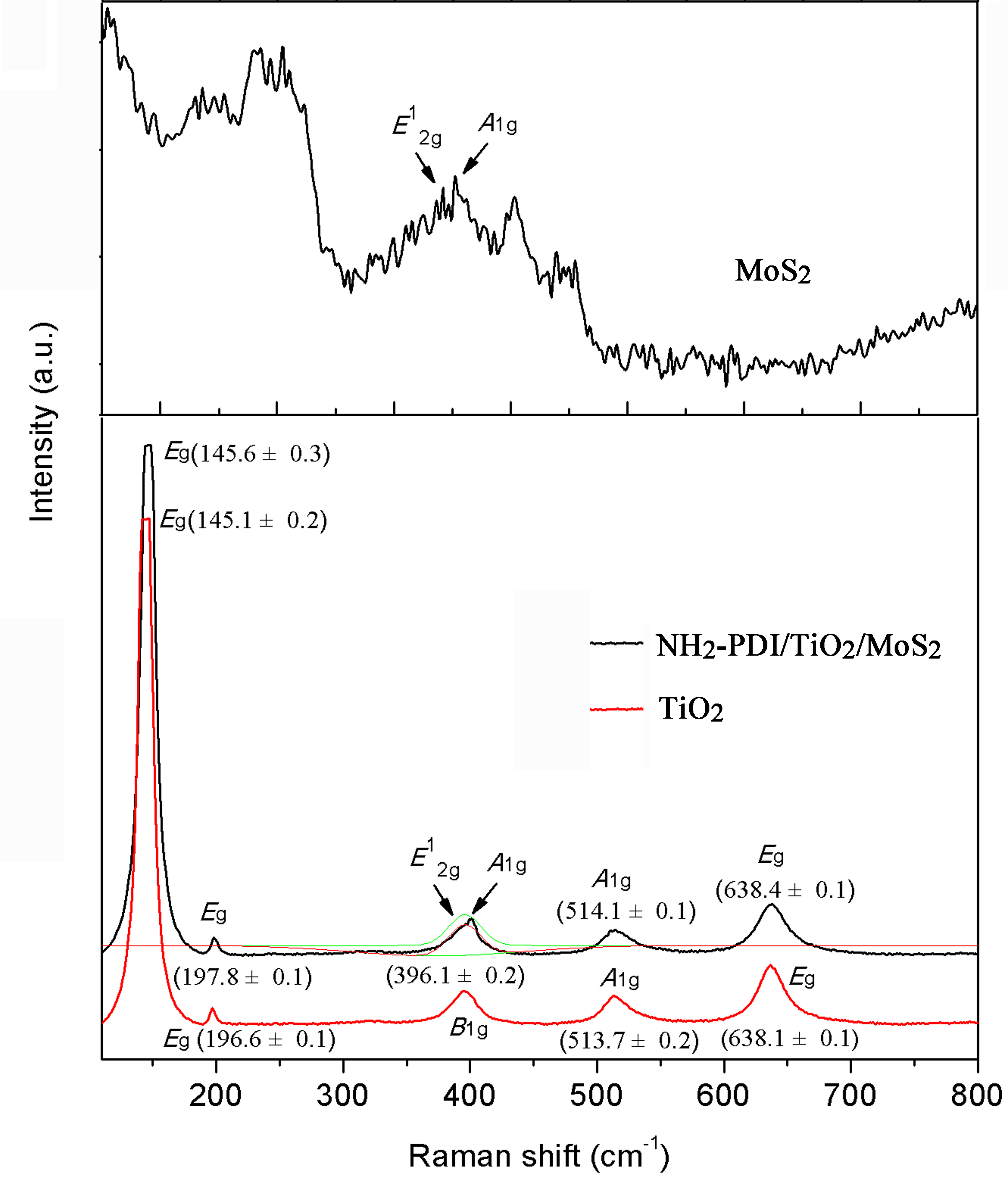
**

Fig. S-4 FTIR spectra of TiO_2_ (a), NH_2_-PDI (b), NH_2_-PDI/TiO_2_ (c), MoS_2_ (d), MoS_2_/TiO_2_ (e), NH_2_-PDI/TiO_2_/MoS_2_ (f).

**
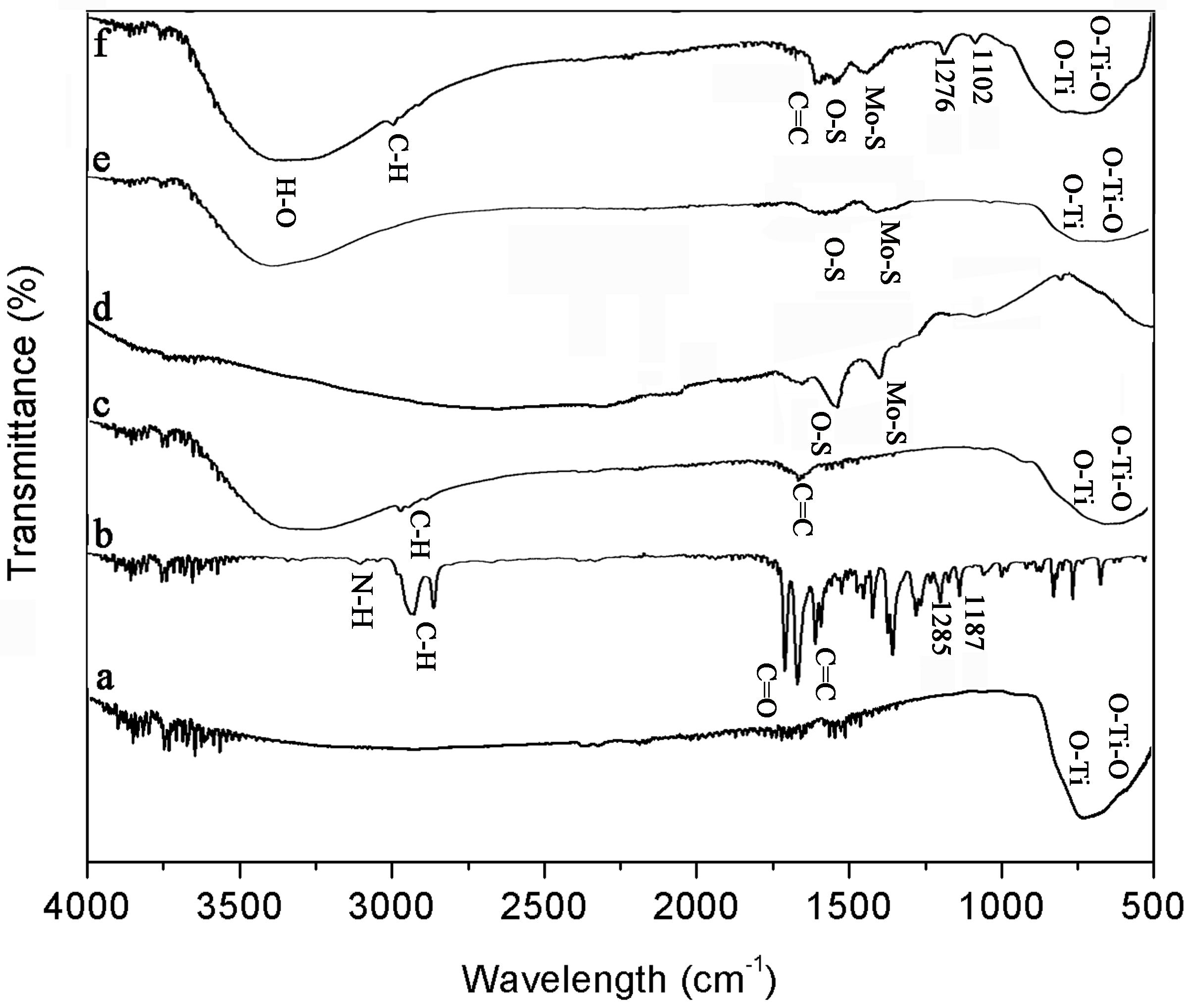
**

Fig. S-5 The absorption spectra obtained in DRS study of samples.


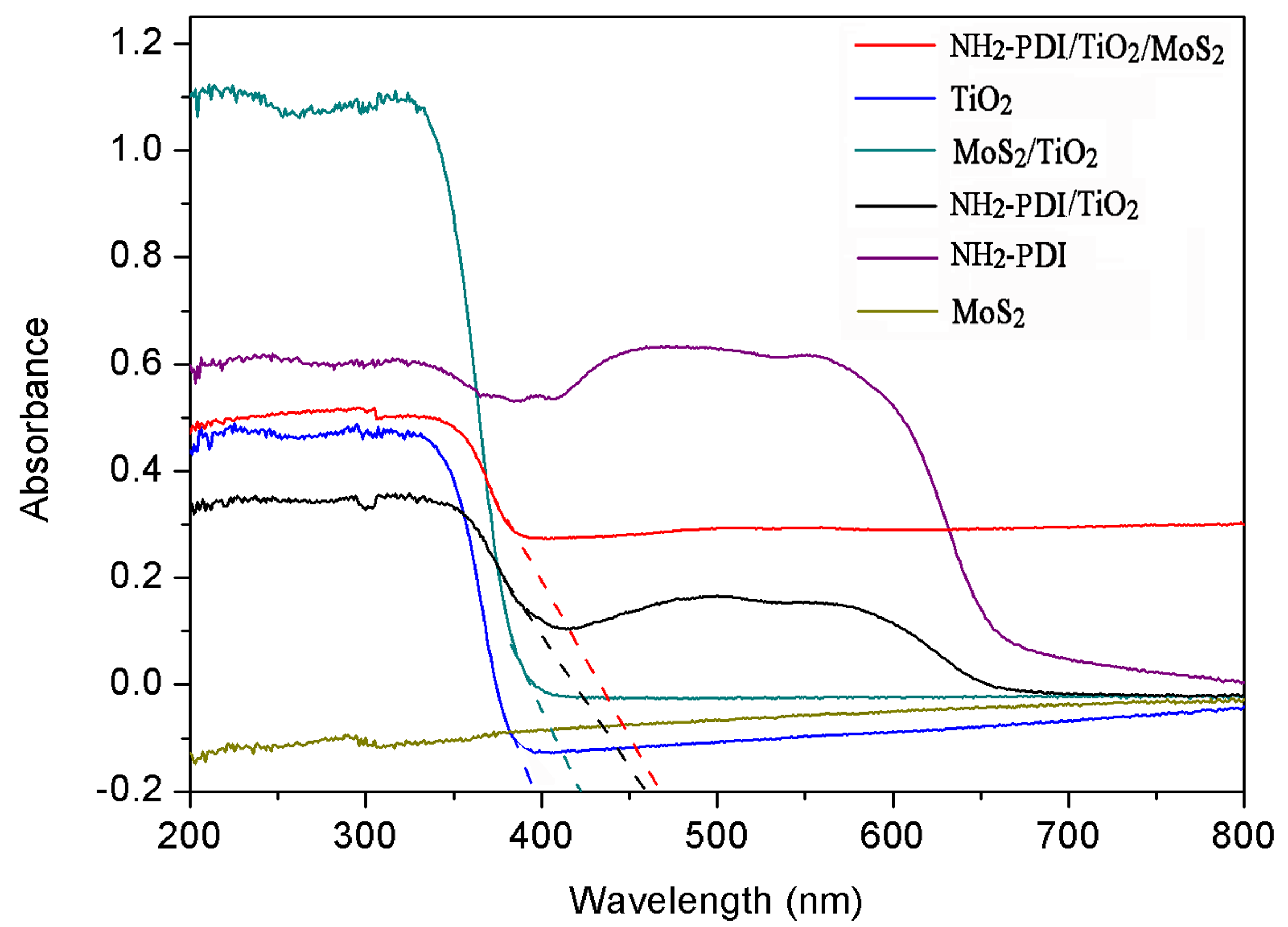


Figure S-6. The changes in UV-vis spectra of MB on irradiation with visible light in the presence of NH_2_-PDI/TiO_2_/MoS_2_. [MB] = 0.01g/L, pH = 7, catalyst suspended = 1g/L.

**
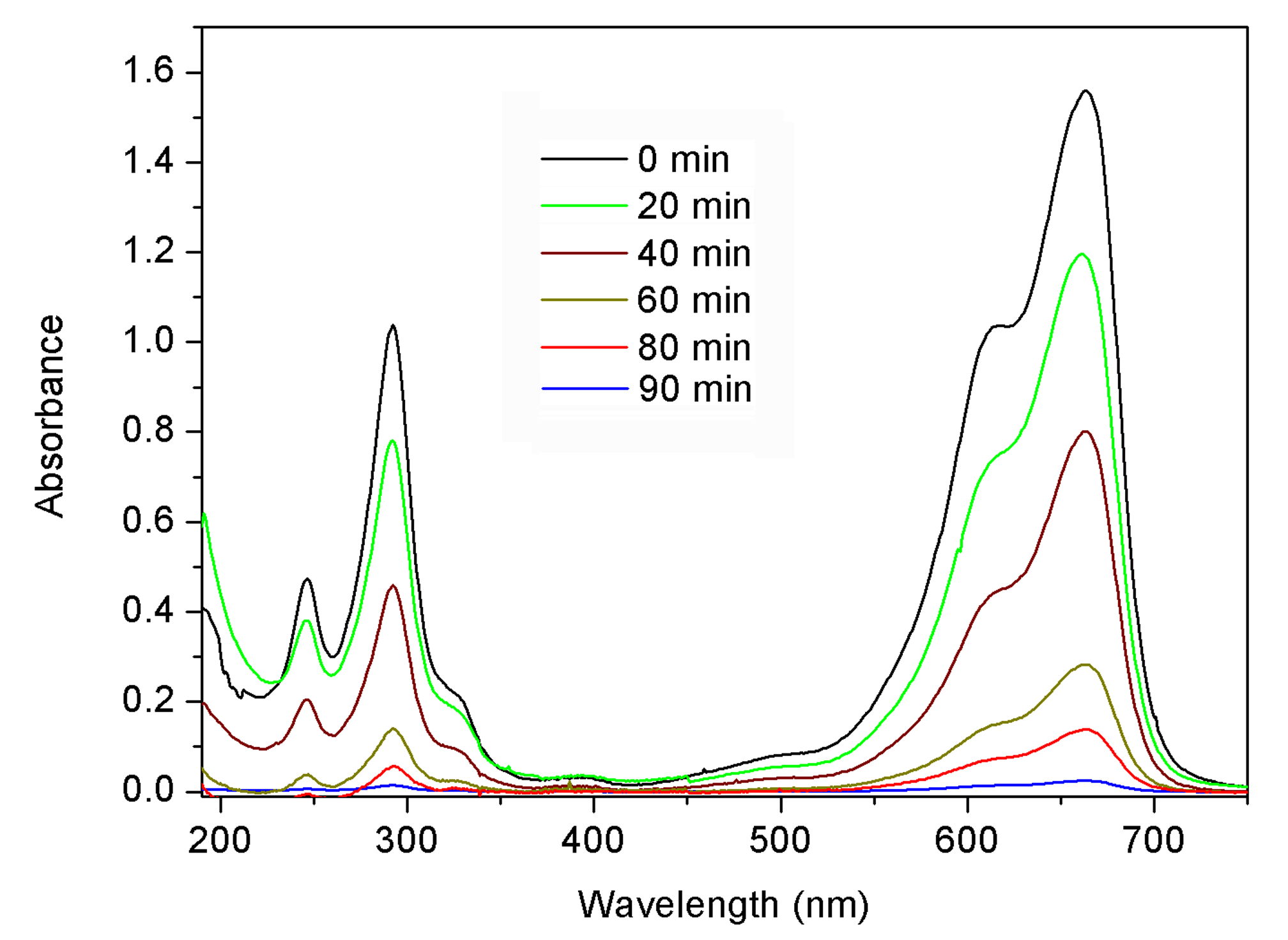
**

Figure S-7. Suggested mechanism for the decolorization of MB.


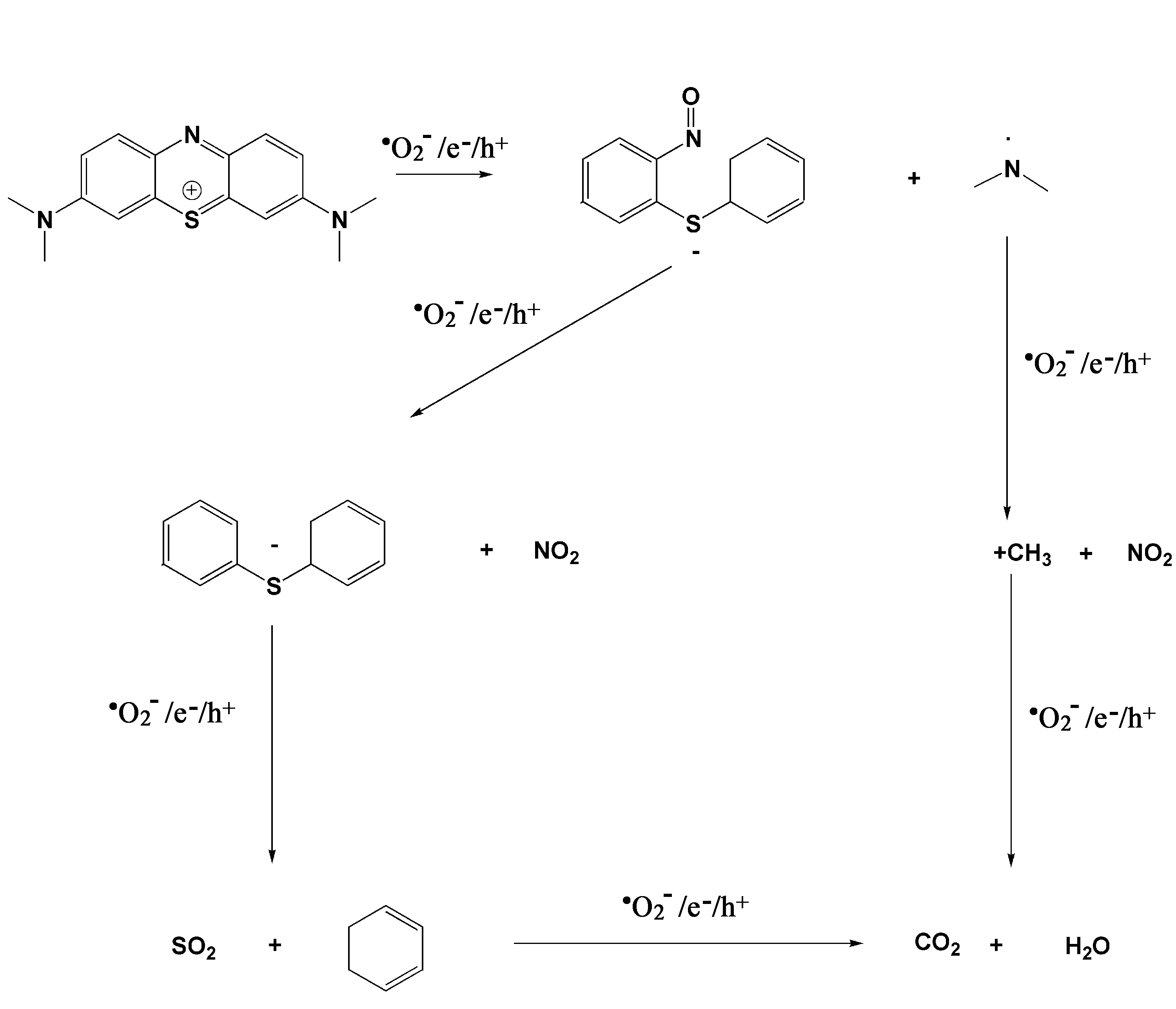


Fig. S-8 Kinetic study of the process based on the results of COD values of MB by catalyst NH_2_-PDI/TiO_2_/2%MoS_2_.


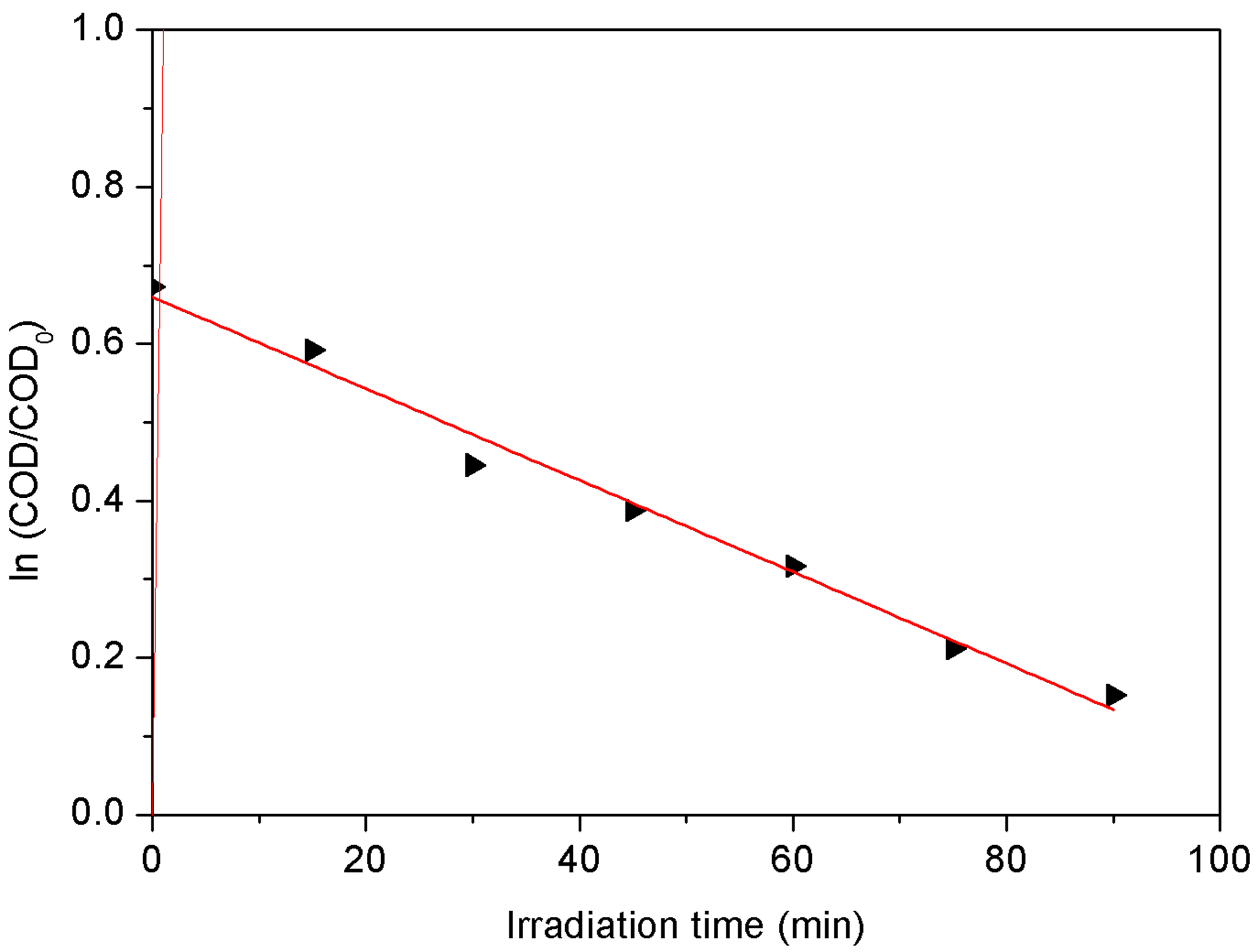


Fig. S-9. (A) The activity diagram of AMX degradation by different catalysts in visible light (A UV-visible spectrophotometer at 198 nm was used to measure the light absorbance of the AMX solution), (B) The first order kinetics curve fitting of AMX degradation by different catalysts. (a) NH_2_-PDI/TiO_2_, (b) MoS_2_/TiO_2_, and (c) NH_2_-PDI/TiO_2_/1%MoS_2_. [AMX] = 0.01g/L, pH = 7, catalyst suspended = 1g/L.


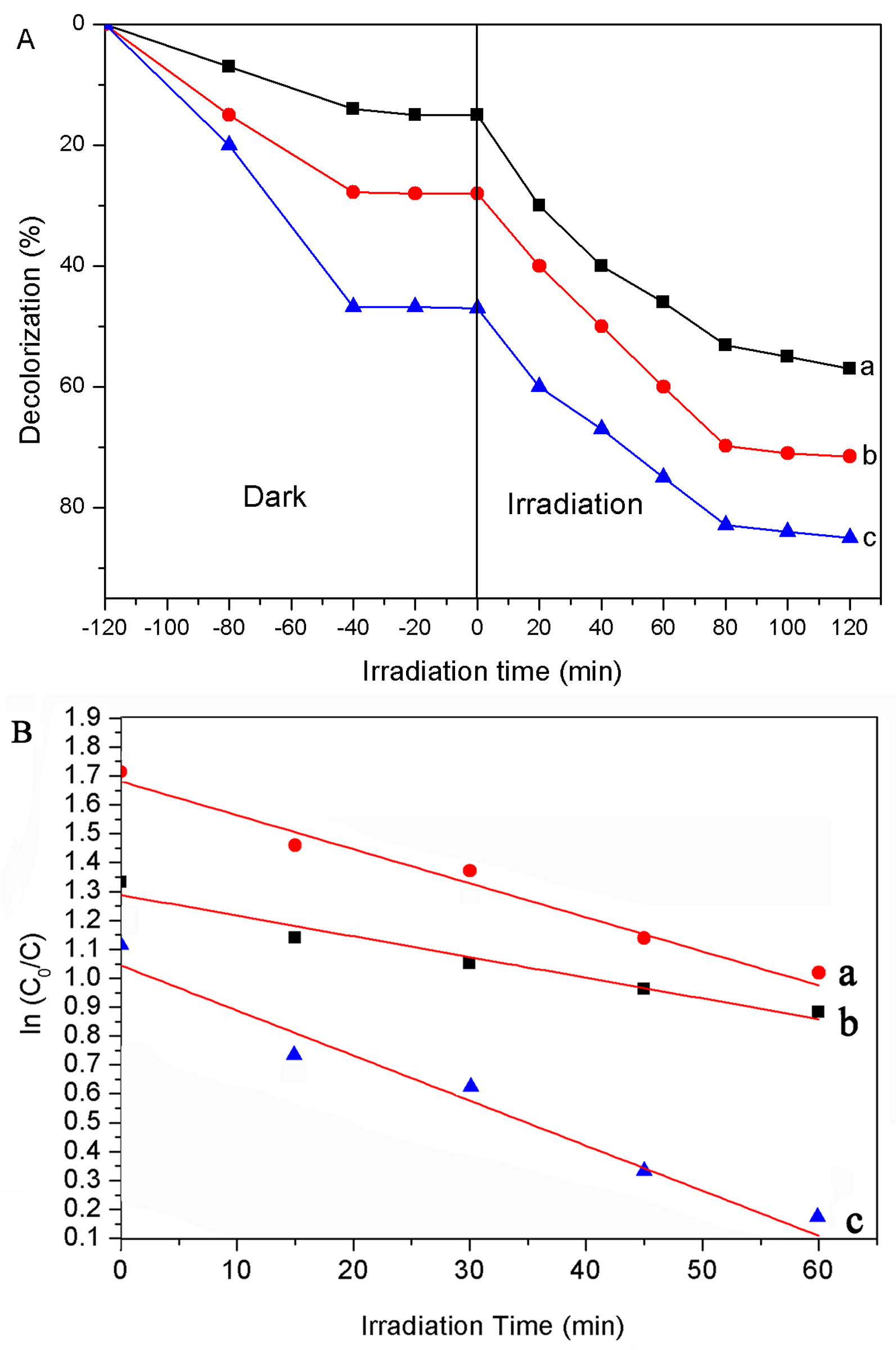


Fig. S-10 (a) Catalyst reusability and (b) FT-IR spectra of NH_2_-PDI/TiO_2_/MoS_2_ photocatalyst before and after 6 cyclicruns under visible light; [MB] = 10 mg/L, pH = 7, catalyst suspended = 1g/L, irradiation time = 120 min.


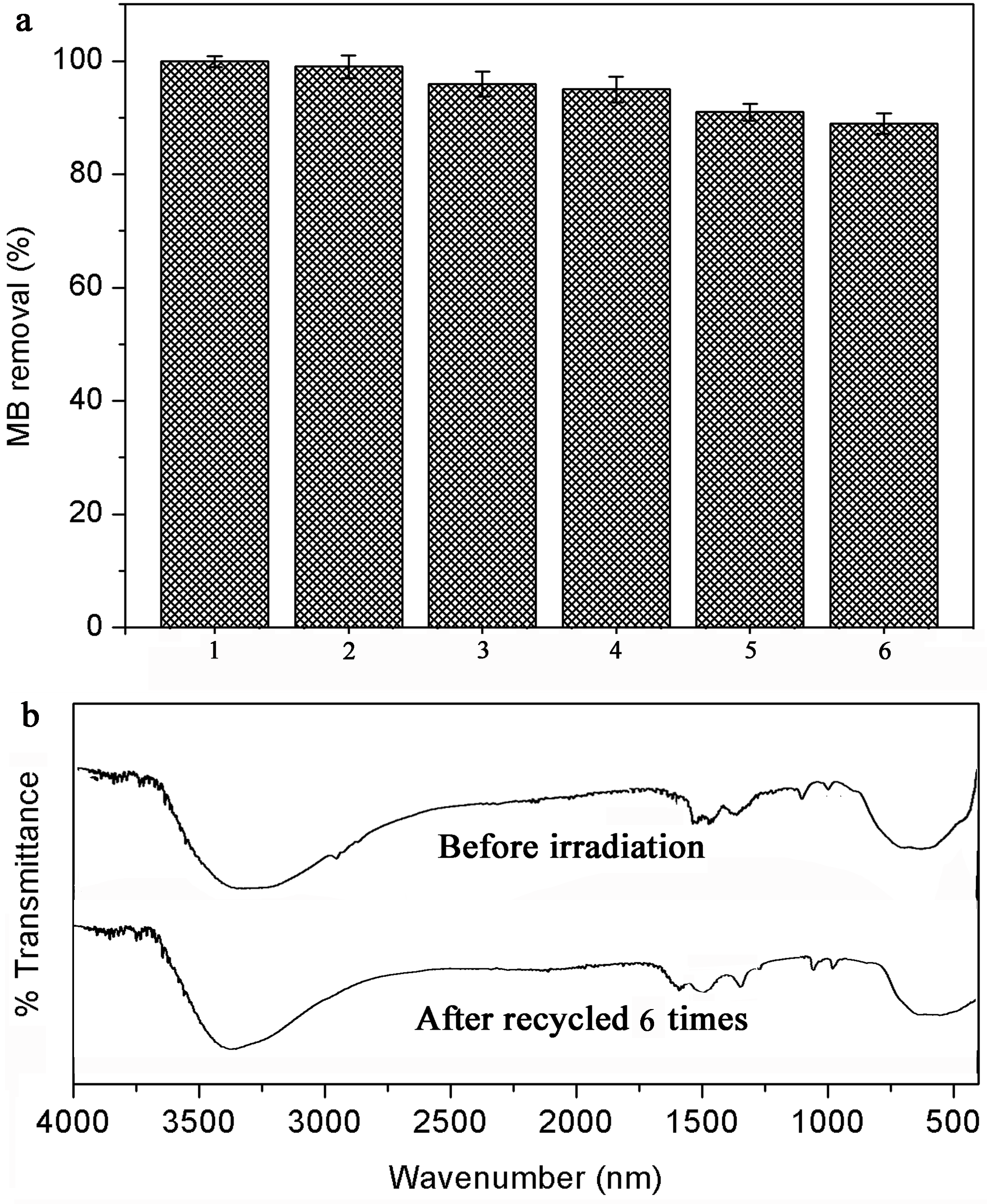


Fig. S-11 Photoluminescence emission spectra of (a) TiO_2_, (b) NH_2_-PDI/TiO_2_, (c) MoS_2_/TiO_2_, and (d) NH_2_-PDI/TiO_2_/MoS_2_ samples. λex= 290 nm.


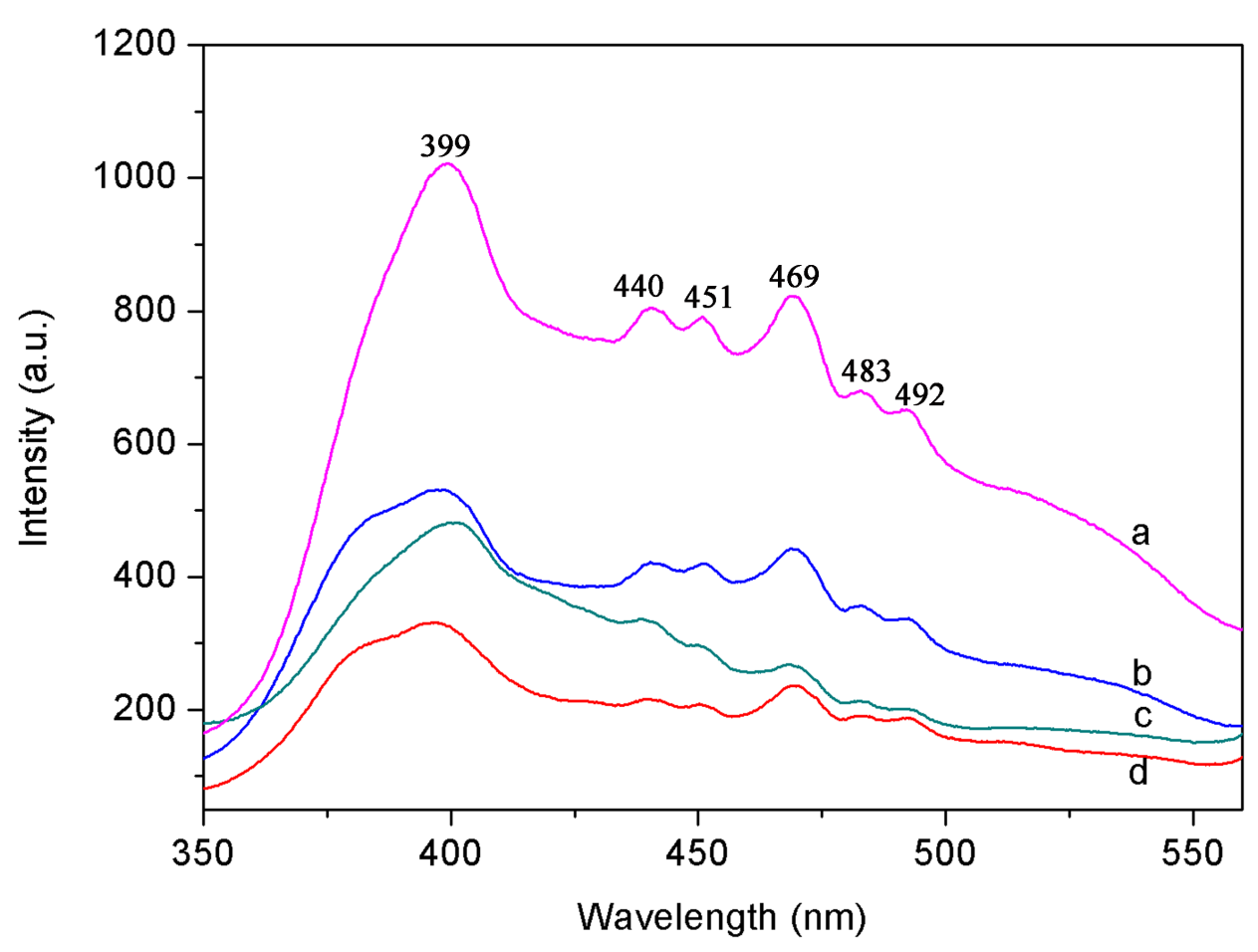


Fig. S-12 The Mott-Schottky plots of pure TiO_2_, NH_2_-PDI and MoS_2_. Counter electrode: Pt. electrolyte: 0.1M Na_2_SO_4_, frequency: 10,000 Hz.


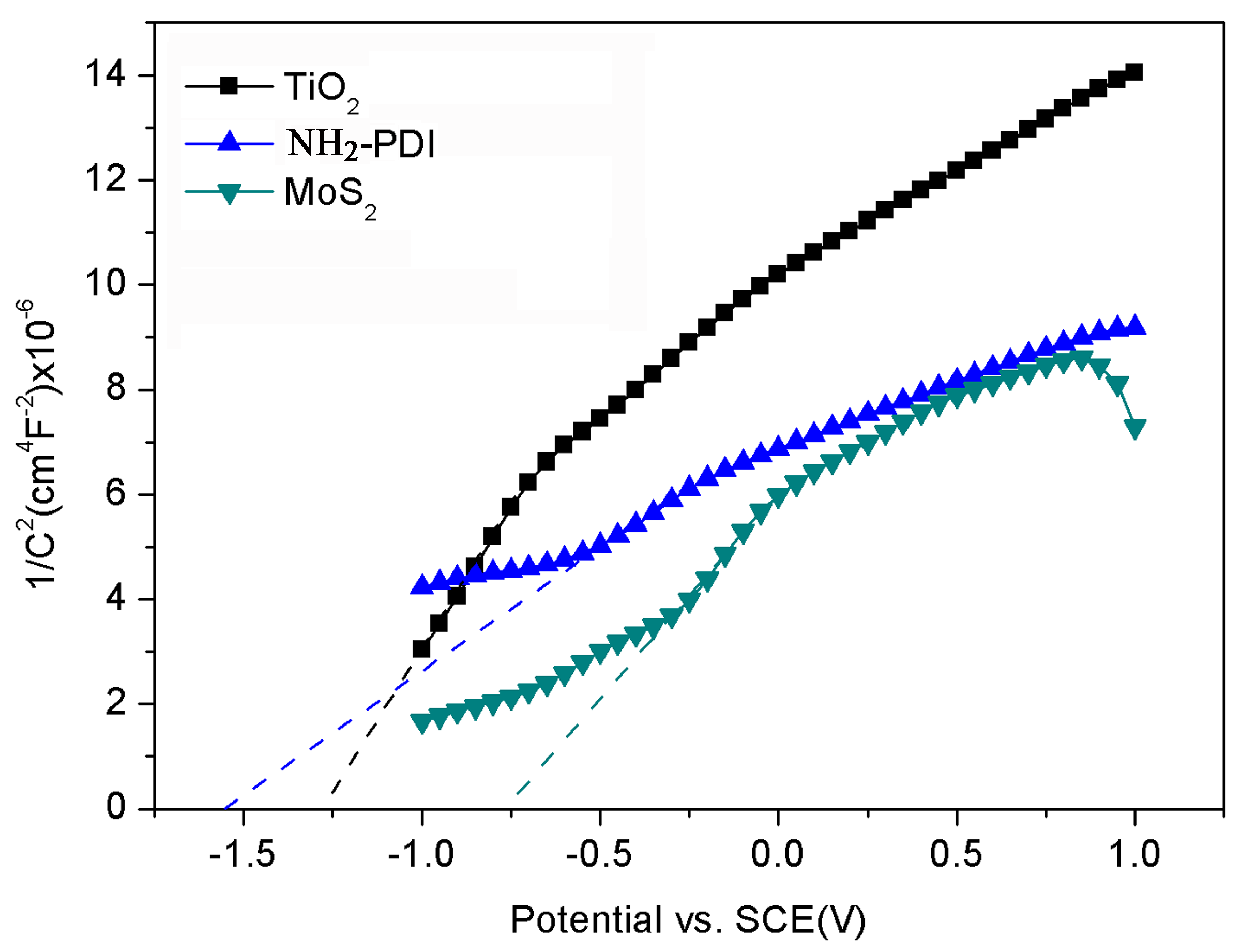


Figure S-13. Plots of photo-generated active species trapped in the system of photodegradation of MB by NH_2_-PDI/TiO_2_/MoS_2_ under visible light.


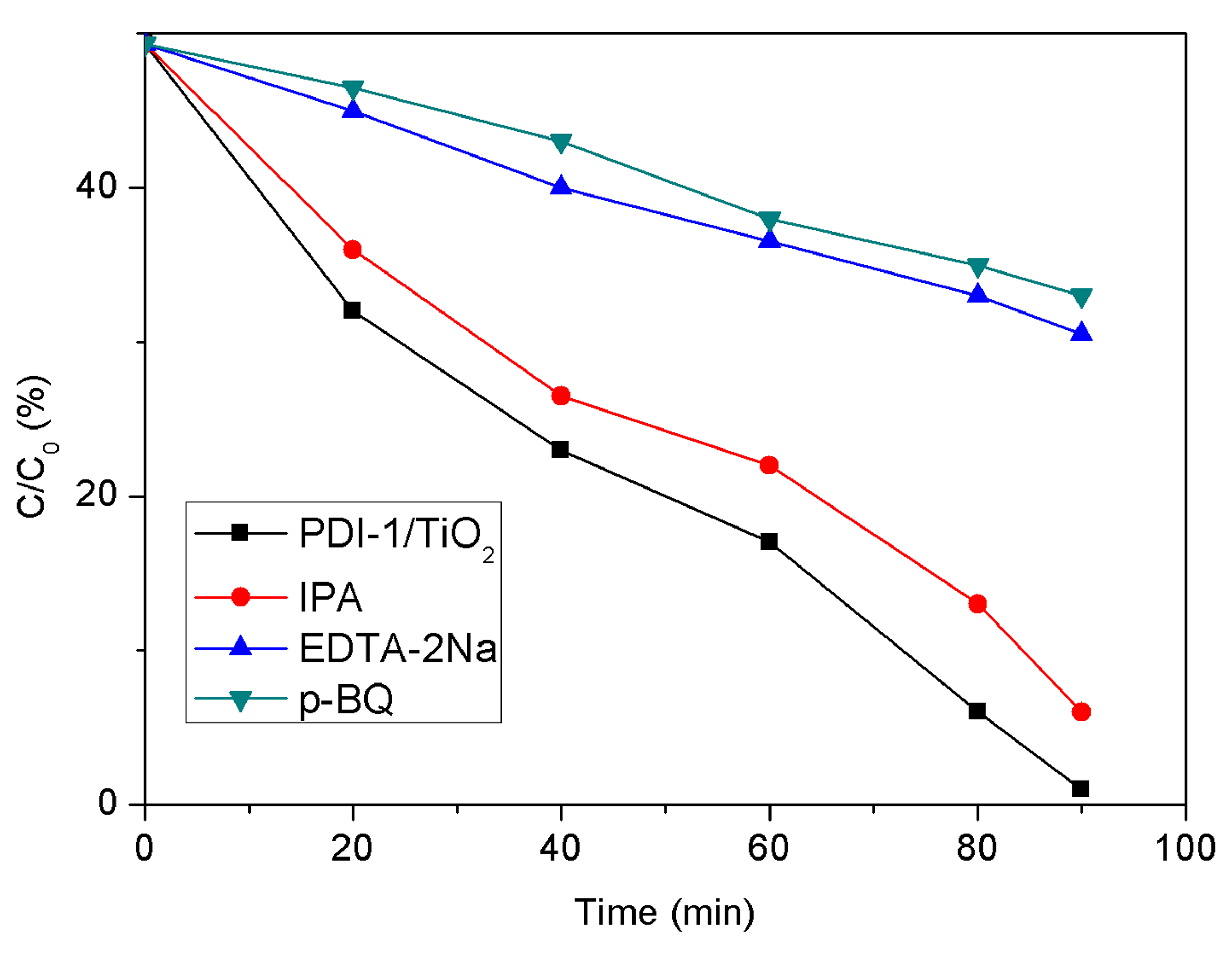

Supplement: Supplementary file 1 — Supplementary Information 1. [file 41598_2020_78894_MOESM1_ESM.doc]
